# Interlayer exciton formation, relaxation, and transport in TMD van der Waals heterostructures

**DOI:** 10.1038/s41377-021-00500-1

**Published:** 2021-04-02

**Authors:** Ying Jiang, Shula Chen, Weihao Zheng, Biyuan Zheng, Anlian Pan

**Affiliations:** 1 Key Laboratory for Micro-Nano Physics and Technology of Hunan Province, School of Physics and Electronics, and College of Materials Science and Engineering, Hunan University, Changsha, China; 2 Wuhan National Laboratory for Optoelectronics, Huazhong University of Science and Technology, Wuhan, China

**Keywords:** Ultrafast photonics, Photonic devices

## Abstract

Van der Waals (vdW) heterostructures based on transition metal dichalcogenides (TMDs) generally possess a type-II band alignment that facilitates the formation of interlayer excitons between constituent monolayers. Manipulation of the interlayer excitons in TMD vdW heterostructures holds great promise for the development of excitonic integrated circuits that serve as the counterpart of electronic integrated circuits, which allows the photons and excitons to transform into each other and thus bridges optical communication and signal processing at the integrated circuit. As a consequence, numerous studies have been carried out to obtain deep insight into the physical properties of interlayer excitons, including revealing their ultrafast formation, long population recombination lifetimes, and intriguing spin-valley dynamics. These outstanding properties ensure interlayer excitons with good transport characteristics, and may pave the way for their potential applications in efficient excitonic devices based on TMD vdW heterostructures. At present, a systematic and comprehensive overview of interlayer exciton formation, relaxation, transport, and potential applications is still lacking. In this review, we give a comprehensive description and discussion of these frontier topics for interlayer excitons in TMD vdW heterostructures to provide valuable guidance for researchers in this field.

## Introduction

**Fig. 1:**
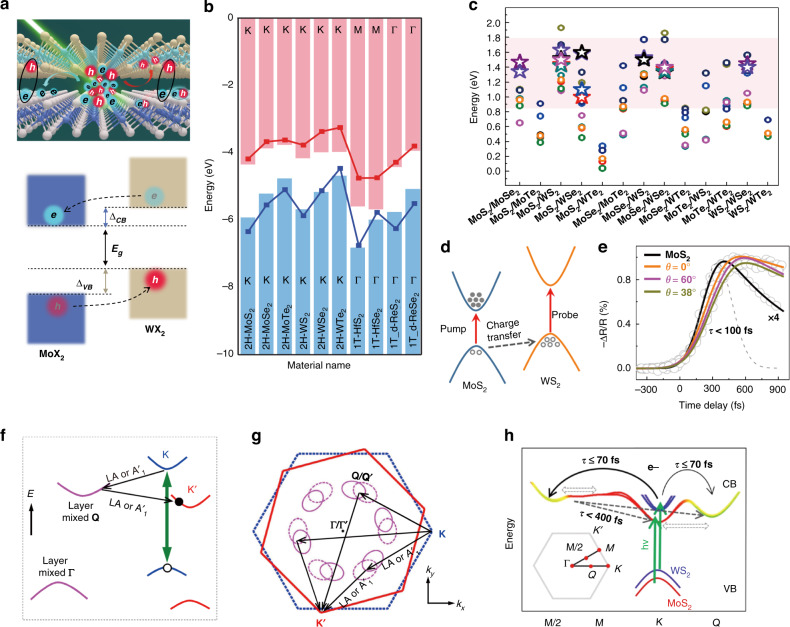
Band alignment and ultrafast charge transfer in TMD vdW heterostructures. **a** Schematic illustration of the side-view structure (top) and the type-II band alignment (bottom) of a TMD vdW heterobilayer. **e/h**: electron/hole; Δ_CB_: conduction band offset of the constituent monolayers; Δ_VB_: valence band offset of the constituent monolayers; *E*_g_: band gap of the heterobilayer. **b** Calculated band alignments of various TMD monolayers^12^. The bar and line-point plots represent the CBM (red color) and VBM (blue color) values obtained from PBE and HSE06 calculations, respectively. The positions of CBM and VBM on the Brillouin zone are shown in the figure. **c** Plot of the calculated band gaps (*E*_g_) and the experimental PL energies of the interlayer excitons versus various TMD vdW heterostructures with a type-II band alignment. The calculated *E*_g_ values (circles) are obtained from references^9,10,12–14^. The experimental PL energies of the interlayer excitons (stars in the pink region) are obtained from references^17,21–23,25,27–29,33,42–45,49,87,90–103^, most of which overlap in the region from ~1.3 to ~1.5 eV. **d** Band alignment of a MoS_2_/WS_2_ heterobilayer^57^. The hole transfers from the VBM of MoS_2_ to that of WS_2_ after optically pumping the MoS_2_ A-exciton. **e** Twist-angle independent charge transfer dynamics (*τ* < 100 fs) obtained by selectively probing the WS_2_ A-exciton of the MoS_2_/WS_2_ heterobilayer^57^. **f**, **g** Schematic illustration of the phonon scattering-mediated interlayer electron transfer process in the energy (**f**) and momentum (**g**) spaces^40^. LA (A_1_′) represents the longitudinal acoustic phonons. **h** Schematic illustration of the ultrafast electron scattering from *K* to *M*, *M*/2, and *Q* valleys within 70 fs (black arrows) in a MoS_2_/WS_2_ heterobilayer and the subsequent electron scattering from M/2 and M back to K′ and Q′ valleys in the other layer within 400 fs (dashed arrows), as evidenced by TR-ARPES^71^. **b** Reprinted with permission from ref. ^12^ [American Physical Society]. **d**, **e** Reprinted with permission from ref. ^57^ [American Chemical Society]. **f**, **g** Reprinted with permission from ref. ^40^ [American Physical Society]. **h** Reprinted with permission from ref. ^71^ [American Physical Society]

Atomically thin transition metal dichalcogenides (TMDs) have received extensive attention due to their unique electronic band structures and the resulting fascinating physical properties^1,2^, such as a direct bandgap in the visible-infrared range, large exciton binding energies of hundreds of meV, and the existence of two intrinsic valley-contrasting quantities, namely, the Berry curvature and the orbital magnetic moment, which allow direct addressing and manipulation of the valley states by external optical, electric, and magnetic fields^3–5^. Furthermore, different TMD monolayers can be vertically stacked to form heterostructures held by weak van der Waals (vdW) forces (Fig. 1a, top panel), which can circumvent the conventional lattice-mismatch problem and thus significantly expand the family of this kind of heterostructure^6^. Additionally, as a new type of quantum material^7,8^, TMD vdW heterostructures not only combine the already extraordinary properties of the constituent monolayers but also provide a rich platform for exploring new fascinating physics and engineering them by various strategies, such as the material type, crystallographic alignment, stacking sequence, or external field.

One such novel physical phenomenon is the emergence of interlayer excitons. Both theoretical^9–14^ and experimental^15–19^ studies demonstrated that most TMD vdW heterostructures feature a type-II band alignment with the conduction band minimum (CBM) and valence band maximum (VBM) located in different monolayers (Fig. 1a, bottom panel), which facilitates interlayer charge transfer, with electrons accumulating in the layer with the lower CBM and holes accumulating in the other layer with the higher VBM. Additionally, the atomic layer thickness of the interlayer separation guarantees strong electron-hole Coulomb interactions between adjacent layers with large binding energies, producing spatially separated but bound electron-hole pairs, that is, interlayer excitons^20–22^. Because of this spatially indirect nature, the reduced overlap of the electron and hole wavefunctions in interlayer excitons provides them with long lifetimes, reaching hundreds of nanoseconds^23–26^ or even microseconds^27^. In addition, the spatial separation between charges creates a permanent electrical dipole moment in the out-of-plane direction, which allows electrical control of their optical and transport properties along with the generation of repulsive dipole–dipole interactions between them^22,28–30^. All these features make interlayer excitons an appealing platform for exploring many-body effects, such as Bose–Einstein condensation (BEC) and superfluidity^31,32^ and highly desirable for developing potential excitonic circuits with long-range exciton transport properties^29,30,33,34^. In addition, the interlayer excitons carry intrinsic valley-contrasting physics inherited from the constituent monolayers, which further enriches fundamental explorations and promising applications with valley functionalities^35^. More intriguingly, the moiré pattern that arises due to the lattice mismatch and rotation angle between the adjacent layers consists of periodically varied local interlayer atomic registries, and is able to create a periodic potential to trap the interlayer excitons with specific optical selective rules, leading to the so-called moiré interlayer excitons, opening up new avenues for quantum manipulation of the quasi-particles towards programmable quantum optics^36^. Apparently, interlayer excitons, as a special excitonic system, bring about a number of fascinating properties that are inaccessible by conventional direct excitons, which lays the basis for exploring novel sciences and developing promising solid-state excitonic devices based on interlayer excitons.

Motivated by these exciting physics from the interlayer excitons in TMD vdW heterostructures, below, we give a systematic overview of interlayer exciton formation, relaxation, transport, and potential applications in excitonic optoelectronic devices. Specifically, the band alignment and ultrafast charge transfer followed by interlayer exciton formation as well as its fundamental properties are first discussed. Moiré interlayer excitons, as a newly emerging popular research topic, are also detailed in this section. Then, the interlayer exciton relaxation processes including the population recombination dynamics, the intervalley scattering process, and the valley-polarized dynamics in TMD vdW heterostructures are reviewed. Later, the interlayer exciton transport under both an electric field and a moiré potential are described. A brief introduction of the recent progress on excitonic optoelectronic devices based on interlayer excitons is given. Finally, we present the conclusions with an outlook on future opportunities for interlayer excitons in TMD-based heterostructures.

## Interlayer exciton formation in TMD vdW heterostructures

In this section, we review the band alignment and charge transfer, interlayer exciton formation, fundamental properties of interlayer excitons, and moiré interlayer excitons in TMD vdW heterostructures.

### Band alignment and charge transfer

The band alignment of semiconductor heterostructures is of central importance in determining their physical properties and potential applications. Three typical types of band alignments (straddling/type-I, staggered/type-II, and broken/type-III) can be constructed in these heterostructures, with their formation being generally understood by Anderson’s rule^14^. For TMD vdW heterostructures, numerous theoretical calculations have been performed to study their band structures, and a type-II alignment was found in most combinations of constituted monolayers (Fig. 1b, c)^9–14^, with the CBM and VBM residing in opposite monolayers. Experimental determination of the band alignments was also carried out and revealed that a staggered form was the prevailing configuration in representative TMD heterostructures. For instance, a type-II alignment with a valence band (VB) offset of 0.83 eV and a conduction band (CB) offset of 0.76 eV was found in the MoS_2_/WSe_2_ heterostructure by using microbeam X-ray photoelectron spectroscopy (μ-XPS) and scanning tunneling microscopy/spectroscopy (STM/STS)^15^. Similarly, a type-II alignment in the MoS_2_/MoTe_2_ heterostructure was measured by using high-resolution XPS and UV–Vis absorption spectroscopy, with a reported VB offset of 0.9 eV and a CB offset of 0.46 eV^19^. The VB offset of 0.3 eV in the MoSe_2_/WSe_2_ heterostructure was also experimentally determined by using submicrometer angle-resolved photoemission spectroscopy (μ-ARPES)^17^. Additionally, the interlayer hybridization, which is sensitive to the interlayer coupling strength (and thus the interlayer distance and twist angle between the constituent monolayers), can affect the band type of TMD vdW heterostructures. Both theoretical^37–41^ and experimental^15,17,18,22,42,43^ results showed that the VBM and CBM of most studied vdW heterostructures favored retention at the *K* valleys in opposite layers to form a direct band gap, with negligible or weak interlayer hybridization near these valleys. However, in the Г and Ʌ (or *Q*) valleys, significant interlayer hybridization was found with an interlayer coupling strength of several hundred meV, which was comparable to the band offset^40^. Such strong interlayer coupling can lead to large energy shifts and may move the VBM/CBM of the heterostructures to the Г/Ʌ (or *Q*) valleys, thus forming an indirect type of band gap in some cases^43–45^, similar to what was observed in homobilayers and bulk TMDs^17,46,47^. In addition to influences from the large differences in band offsets at various valleys, the difference in the orbital characteristics was considered an important factor for the resulting variant interlayer hybridization in momentum space^17,48^. It was reported that the bands at *K* valleys generally featured an in-plane orbital character, while those at Г/Ʌ (or *Q*) valleys were characterized by an out-of-plane orbital character and therefore were more sensitive to the interlayer interactions for orbital hybridization^48^. Hence, the interlayer distance and twist angle (or stacking manner), which affect the interlayer interactions and/or the interlayer coupling strength, could substantially modify the bands at the Г/Ʌ (or *Q*) valleys. This was supported by the fact that the energies of the Г and Ʌ (or Q) points depended sensitively on the interlayer distance and stacking manner^37,42,49–51^.

Following both theoretical predictions and experimental determination of a type-II band alignment in most TMD vdW heterostructures, charge transfer as a natural outcome of this configuration spurred research interest in these two-dimensional (2D) heterostructures^52–54^. The first experimental observation of ultrafast charge transfer was reported in a MoS_2_/WS_2_ heterostructure using both photoluminescence (PL) mapping and femtosecond pump-probe spectroscopy^52^. This demonstrated that holes in the MoS_2_ monolayer could efficiently transfer into the WS_2_ monolayer within 50 fs after photoexcitation. Then, both electron and hole transfer in opposite directions on a sub-picosecond time scale were evidenced in the MoS_2_/MoSe_2_ heterostructure^53^. After these initial works, charge transfer dynamics in TMD vdW heterostructures have been extensively investigated, from which the interfacial charge transfer was found to be universally ultrafast (mostly within 100 fs) and twist-angle independent (Fig. 1d, e)^42,55–63^. These observations are very puzzling because (1) the interlayer van der Waals coupling in heterostructures is normally much weaker than the intralayer covalent bonding, and hence, the interlayer charge transfer is not expected to be so rapid as compared to the intralayer exciton dynamics; and (2) momentum mismatch in displaced ±K/±K′ valleys is inevitable due to the lattice mismatch and twist-angle between the constituent monolayers (note that “+K” and “−K” (or “+K′” and “−K′”) indicate the opposite valleys of the same monolayer, while “+K” and “+K′” (or “−K” and “−K′”) describe the same *K* valley but in the opposite monolayers. The same applies to other valleys such as the *Q* and Г valleys described below), so how can the interlayer charge transfer be so effective and twist-angle independent, regardless of the crystal orientation or momentum mismatch?

A number of studies have been carried out to explore the intrinsic mechanism for efficient charge transfer in TMD vdW heterostructures^38,40,64–72^. In TMD monolayers, one may find that the exciton binding energy (0.5–1 eV) is comparable to the typical Frenkel exciton^73–77^, while its wavefunction favors a Wannier–Mott type with electron-hole separation extending over several tens of unit cells (the exciton Bohr radius was calculated to be ~1–3 nm)^73,78,79^. Therefore, for TMD vdW heterostructures, although the constituent monolayers are in contact with weak vdW coupling, the layer separation is less than 1 nm, and the layer-separated electrons and holes can still undergo strong Coulomb interactions to form bound exciton states^2,35,52^ (i.e., interlayer excitons, which are discussed later). These bound exciton states are thought to be energetically favorable and can compete with the intralayer exciton states^52^. That is, the photoexcited electrons and holes should have comparable probability to form these layer-separated bound exciton states in addition to the intralayer exciton states, which probably accounts for the efficient interlayer charge transfer in vdW heterostructures^52^. For the observed twist-angle independence of charge transfer^56–58^, a very likely mechanism is that phonon scattering coupled with interlayer hybridization circumvents the momentum mismatch for such rapid charge transfer^40^. As proposed (Fig. 1f, g)^40^, interlayer charge transfer can efficiently take place via two sequential steps: first, a photoexcited electron is scattered from the *K* valley to the strongly layer hybridized *Q*/*Q*′ valley (Г/Г′ valley for a hole) through the emission of an intralayer phonon, and then it subsequently relaxes from the *Q*′ valley (Г′ valley for a hole) to the *K*′ valley in the opposite layer by emitting another phonon. The interlayer charge transfer via *Q*/*Q*′ or Г/Г′ valleys is expected to be rapid (<50 fs) due to the strong interlayer coupling or hybridization in these regions. Moreover, the Г positions with strong layer mixing are not affected by the interlayer twist, and the *Q* valleys are always on a ring region with strong interlayer coupling for any twist angle (Fig. 1g), both of which explain the observed twist-angle independence of the charge transfer well. This proposed mechanism was recently experimentally supported in a MoS_2_/WS_2_ heterobilayer with time-resolved and angle-resolved photoemission spectroscopy (TR-ARPES)^71^, where the ultrafast scattering of electrons from the *K* to *M*, *M*/2, and *Q* valleys was evidenced within 70 fs, followed by electron scattering from *M*/2 and *M* back to the *K*′ and *Q*′ valleys in the other layer within 400 fs (Fig. 1h). These studies suggest that Г and *Q* valleys with strong interlayer hybridization play an important role in mediating ultrafast and twist-insensitive charge transfer in TMD vdW heterostructures.

### Interlayer exciton formation

As stated above, immediately after rapid interlayer charge transfer, a strong Coulomb interaction of the electrons and holes in opposite layers could exist due to the layer separation (~0.7 nm) comparable to the intralayer exciton Bohr radius (~1–3 nm)^73,78,79^, which facilitates the formation of interlayer excitons that have been both theoretically^80^ and experimentally evidenced^20–22,42^. The binding energy of the interlayer exciton, with a reported value of ~100–350 meV from both theoretical^81–86^ and experimental^17,21,42,55,87–89^ results, is a further indication of the strong Coulomb interaction strength between the layer-separated electrons and holes, of which the value depends sensitively on the interlayer distance^83,85^.

**Fig. 2:**
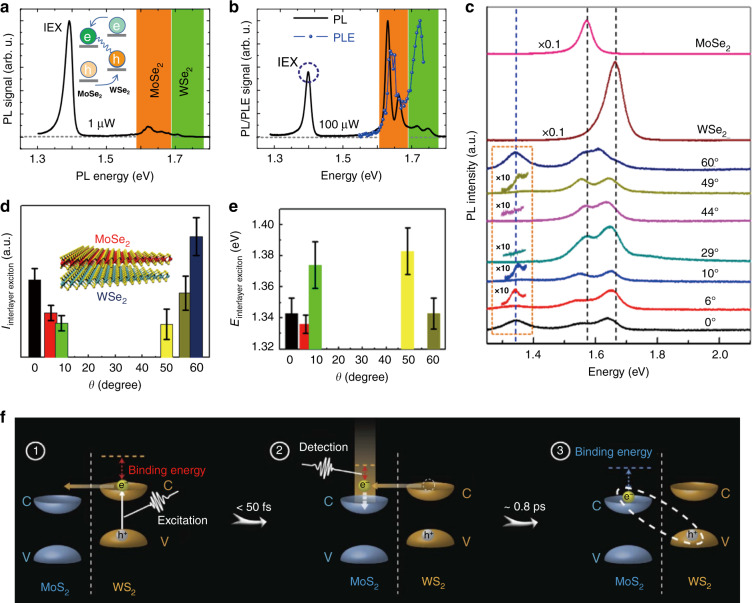
Interlayer exciton formation in TMD vdW heterostructures. **a** PL spectrum of the MoSe_2_/WSe_2_ heterostructure measured at 4.5 K with 1 μW excitation power^28^. IEX represents the emissions from interlayer excitons. **b** PL spectrum of the MoSe_2_/WSe_2_ heterostructure (black line) measured at 4.5 K with 100 μW excitation power and the PLE spectrum (blue dots) of the interlayer exciton emission (IEX, marked by the blue dashed circle)^28^. **c** PL spectra of the MoSe_2_ monolayer, WSe_2_ monolayer, and MoSe_2_/WSe_2_ heterobilayer for various twist angles (0° ≤ *θ* ≤ 60°) at room temperature^101^. **d**, **e** Twist-angle-dependent PL intensity (**d**) and energy (**e**) of the interlayer excitons formed in MoSe_2_/WSe_2_ heterobilayers^101^. **f** Illustration of the interlayer exciton formation process in MoS_2_/WS_2_ heterostructures as revealed by transient absorption spectroscopy^55^. **a**, **b** Reprinted with permission from ref. ^28^ [IOP Publishing]. **c**–**e** Reprinted with permission from ref. ^101^ [American Chemical Society]. **f** Reprinted with permission from ref. ^55^ [Springer Nature Limited]

The resulting interlayer excitons feature the lowest energy configuration, with the electrons and holes residing in the CBM and VBM of the opposite layers due to the type-II band alignment. Hence, the hallmark of forming an interlayer exciton is the appearance of an extra PL peak at a lower energy along with PL quenching of the constituent monolayers (Fig. 2a), which has been observed in various representative TMD vdW heterostructures such as WS_2_/WSe_2_^58,90,91^, MoS_2_/MoSe_2_^53,87,92^, MoS_2_/WS_2_^42,43,49,55,93,94^, MoS_2_/WSe_2_^20,21,29,44,95–97^, MoSe_2_/WS_2_^98–100^, MoSe_2_/WSe_2_^17,22,23,25,27,28,33,45,101–104^, and MoS_2_/MoSe_2_/MoS_2_^24,105^ and also in homobilayers^46,106^, with the PL energy of interlayer excitons ranging from ~1.0 to ~1.6 eV (Fig. 1c). PL excitation (PLE) measurement is an alternative tool for supporting interlayer exciton formation^22,24,28^. As observed (Fig. 2b)^28^, the PL emission of interlayer excitons only emerges when the laser energy is high enough to excite the monolayer with a narrower band gap, and the PL intensity is resonantly enhanced when the laser energy matches the A-exciton resonance of each monolayer, suggesting the interlayer nature of such an emission. The formation dynamics of the interlayer excitons have also been revealed by various transient spectroscopies^55,89^. In a MoS_2_/WS_2_ heterostructure^55^, the interlayer exciton formation process was reported to include two steps (Fig. 2f): first, an intermediate hot interlayer exciton state with a binding energy <0.17 eV was initially formed within 50 fs, and second, it relaxed to a tightly bound interlayer exciton state within ~800 fs featuring a binding energy >0.36 eV. However, a recent work demonstrated that the interlayer exciton state in the WS_2_/WSe_2_ heterostructure^89^, with a binding energy of ~0.13 eV, was formed within 100 fs and directly transformed from the intralayer exciton gas without a pronounced intermediate hot state. The origin of these discrepancies in the formation process of interlayer excitons needs to be further explored.

Note that interlayer excitons are not always formed or observed in vdW heterostructures. Their emergence requires certain prerequisites such as appropriate interlayer distance and momentum mismatch, both of which could significantly affect the interlayer coupling between the constituent monolayers. The interlayer distance of the heterobilayers is generally improved by thermal annealing^49,92,100^, and can be controlled by inserting insulate layers (i.e., hBN) between the TMD monolayers^20,107^. The momentum mismatch is predominantly influenced by the lattice mismatch and twist angle of the opposite monolayers. In contrast to the interlayer charge transfer, both the PL intensity and energy of the interlayer excitons were demonstrated to have twist-angle dependence^41,42,95,100,101^. For instance, in the MoSe_2_/WSe_2_ heterostructures^101^, the PL intensity of the interlayer excitons was enhanced at angles near 0° and 60° but disappeared at other intermediate angles (10–50°), and their PL energies also varied with the twist angle (Fig. 2c–e), which was attributed to the twist-angle dependence of the interlayer coupling strength in these heterostructures. In addition, it was reported that the PL energy of interlayer excitons could be tuned by many other factors such as the vdW bandgap^90^, electric and magnetic fields^22,108–110^, cavity^111–113^, pressure^114^, and layer number^115^. Moreover, even electroluminescence (EL) from interlayer excitons was observed in TMD vdW heterobilayers under a forward bias^30,32,102^.

### Fundamental properties of interlayer excitons

An interlayer exciton is an exotic exciton system that is distinguished from an intralayer exciton and possesses various novel and intriguing properties, such as the existence of a reduced transition dipole and a static electric dipole as well as inherited valley-contrasting physics from monolayers. All these properties are of central interest in both fundamental physics and potential applications, as discussed below.

#### Reduced transition dipole

**Fig. 3:**
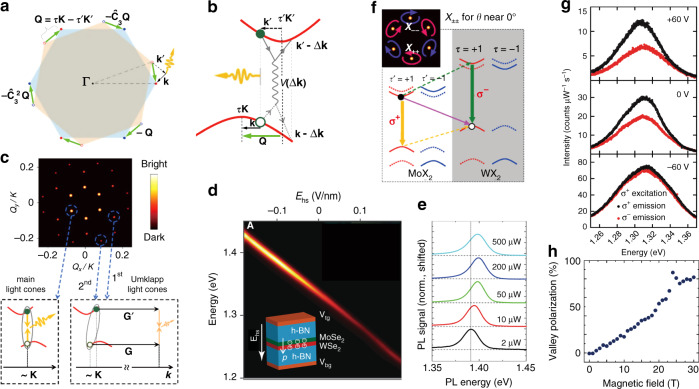
Fundamental properties of interlayer excitons. **a** Brillouin zone corners of the WX_2_ layer (τ**K**, the solid dots) and the MoX_2_ layer (τ′**K**′, the open dots) with a small twist and/or lattice mismatch^80^. The green arrow represents the displacement vector between the τ**K** and τ′**K**′ corners of the constituent layers. $\hat C_3$ is the three-fold rotational symmetry. **b** The electron-hole interlayer Coulomb interaction (*V*(Δ***k***)) conserves their kinematical momentum sum **Q**^80^. The interlayer exciton with a certain kinematical momentum **Q** equaling the momentum mismatch between the electron and hole (**Q** = **τK−τ**′**K**′, the green arrows) can recombine to emit a photon. **c** The main, first-Umklapp, and second-Umklapp light cones in **Q** space for MoSe_2_/WSe_2_ heterobilayers with twist angles *θ* near 0° or 60°^80^. **d** PL energy of the interlayer exciton versus the electric field (*E*_hs_) applied on the MoSe_2_/WSe_2_ heterostructure^30^. The inset schematically displays the heterostructure cross-section. The white arrows represent the directions of the electric field (*E*_hs_) and the static electric dipole (*p*). **e** PL spectra of the interlayer excitons in MoSe_2_/WSe_2_ heterostructures measured at 4.5 K as a function of excitation power^28^. **f** Theoretically predicted valley-dependent elliptically polarized optical selection rules for interlayer excitons (*X*_−−_, *X*_++_) in the six main light cones^80^. The dipole transition (interlayer hopping) is denoted by solid (dashed) arrows. The valley indices (*τ*, *τ*′) correspond to (+, +) or (−, −) for MoX_2_/WX_2_ heterobilayers with twist angle *θ* near 0°. **g** Circular polarization-resolved PL spectra of the interlayer exciton at selected gate voltages. All the data were obtained under σ+ circularly polarized light excitation, with the co-polarized (σ+) and cross-polarized (σ−) PL spectra shown in black and red, respectively^33^. **h** Magnetic-field-dependent valley polarization of the interlayer exciton^25^. **a**–**c**, **f** Reprinted with permission from ref. ^80^ [American Physical Society, Springer Nature Limited]. **d** Reprinted with permission from ref. ^30^ [American Association for the Advancement of Science]. **e** Reprinted with permission from ref. ^28^ [IOP Publishing]. **g** Reprinted with permission from ref. ^33^ [American Association for the Advancement of Science]. **h** Reprinted with permission from ref. ^25^ [Springer Nature Limited]

Due to the spatially indirect nature of the interlayer exciton, its transition dipole was theoretically predicted to be one to two orders of magnitude smaller than that of the intralayer exciton^80,116^, and therefore, the corresponding oscillator strength is expected to be dramatically reduced with respect to that of an intralayer exciton. This is supported by an experimental study of the photocurrent measurements of interlayer excitons in a MoSe_2_/WSe_2_ heterobilayer^102^, where the photocurrent amplitude from the interlayer excitons was shown to be approximately 200 times smaller than that of the intralayer excitons, meaning that the interlayer exciton oscillator strength was two orders of magnitude smaller than that of the intralayer excitons due to the spatial separation of the electrons and holes in the opposite layers. In addition, the transition dipole of the interlayer excitons was shown to be quite sensitive to the kinematical momentum sum (**Q**) of the electrons and holes in the opposite monolayers, leading to so-called light cones with an ordered hexagonal array in momentum space (Fig. 3a–c)^35,80^. As shown in Fig. 3b, an interlayer exciton consists of an electron situated at the momentum position *τ*′**K**′ + **k**′ and a hole at *τ***K** − **k** (*τ*′ and *τ* are the electron and hole valley indices)^35,80^. The kinematical momentum (**Q**) of the interlayer exciton (or the kinematical momentum sum of the electron and hole) was defined as **Q** ≡ **k**′ + **k** by previous reports^35,80^. For an interlayer exciton with zero kinematical momentum (**Q** = 0), the electron and hole are located separately in the CBM (*τ*′**K**′) and VBM (*τ***K**) with uncompensated momentum mismatch due to the unavoidable lattice mismatch and/or interlayer twist between the layers, and in this case, the interlayer exciton is considered to be optically dark with a vanished transition dipole and forbidden direct radiative recombination; namely, the interlayer exciton is out of the light cone. However, for an interlayer exciton with a certain kinematical momentum **Q** by which the momentum mismatch between the electron and hole can be compensated (that is, **Q** = τ**K** − *τ*′**K**′), in this case, the interlayer exciton possesses a finite transition dipole for the direct optical transition and thus is optically bright; namely, the interlayer exciton is within the light cone. In addition, the transition dipole strength of a light cone was reported to decay quickly with the magnitude of the momentum^35,80^, so the most bright light cones are those at **Q** values nearest to **Q** = 0, and other light cones are Umklapp type (Fig. 3b), which is consistent with experimental results showing that the interlayer excitons can only be observed in heterostructures with relatively small interlayer distances and limited rotational angles for the finite interlayer coupling between layers. Compared to an intralayer exciton, a significant merit of the reduced transition dipole of an interlayer exciton is its substantially extended recombination and valley polarization lifetimes due to the reduced overlap of the electron and hole wavefunctions, which inspires research exploring spin-electronic, valley-electronic, and optoelectronic physics and applications based on TMD vdW heterostructures.

#### Static electric dipole

Since electrons and holes are confined in opposite layers of a type-II vdW heterostructure, interlayer excitons have a static electric dipole along the out-of-plane direction (*p* = *e* · *d*, where *e* is the charge quantity and *d* is the charge separation distance), which allows their energy to be tuned (Δ*ε*) by an external electric field (*E*) along the dipole axis (i.e., the Stark effect, Δ*ε* = −*p* · *E*)^22,95,117^. Strong and linear tuning of the interlayer exciton PL energy with an applied electric field was indeed observed in several studies with a tuning range of ~80–138 meV^22,26,30,86,95,117^, reflecting that the linear Stark effect mainly contributed to the energy shift (Fig. 3d). From the linear fit of the energy shift versus electric field, the dipole size or charge separation (*d*) has been estimated to be ~0.5–0.8 nm, matching well with the expected layer separation (~0.7 nm)^30,95,117^. Another consequence of the static electric dipole is the resulting dipole–dipole repulsive interactions between the interlayer excitons^22,28,30,118,119^, as revealed by a blue-shift in the PL energy with increasing exciton density (excitation power) (Fig. 3e)^22,28,30^. Such repulsive interactions owing to the static electric dipole, combined with the ultralong lifetimes due to the reduced transition dipole, make interlayer excitons a highly promising platform for exploring excitonic Bose–Einstein condensate (BEC) and superconductivity phenomena^31,32,120^.

#### Valley-contrasting physics

The valley pesudospins in TMD monolayers are known to be accessible and controllable by external optical fields (via valley-dependent optical selection rules)^121–124^, electric fields (via the valley Hall effect)^125–128^, and magnetic fields (via the valley Zeeman effect)^129,130^ owing to the existence of intrinsic valley-contrasting physical quantities (namely, the Berry curvature and orbital magnetic moment) to distinguish the ±*K* valleys^131–133^. It was reported that such valley-contrasting physics were inherited by the interlayer excitons in TMD vdW heterostructures^80,104^. Theories predicted that interlayer excitons in the light cones have valley-dependent optical selection rules with opposite helicities for opposite valleys (Fig. 3f)^80,134^, which was experimentally evidenced in circularly polarized PL measurements with apparent valley polarization for the interlayer excitons^24,27,33,45,117^. For instance, a valley polarization up to 80% was reported for interlayer excitons in a MoSe_2_/WSe_2_ heterostructure (without externally applied electric or magnetic fields)^135^. Such high valley polarization of interlayer excitons implies efficient interlayer charge transfer accompanied by conserved spin–valley polarization transfers, as revealed in the MoSe_2_/WSe_2_ heterostructure, with robust spin–valley conserved transfers for different twist angles^104^. Additionally, the valley polarization of interlayer excitons can be manipulated by external electric and magnetic fields, with the polarization degree varying with the applied field (Fig. 3g, h)^25,27,33,110,117^. Moreover, both valley Hall and Zeeman effects were observed for interlayer excitons^25,110,136^, similar to those for intralayer excitons. All of these results indicate that the valley-contrasting physics in monolayers is successfully inherited by the interlayer excitons. However, note that circularly polarized PL from interlayer excitons with a helicity opposite to the optical excitation was also observed in TMD vdW heterostructures^24,137^, implying a reversed polarization selection rule for interlayer excitons in certain cases. In addition, due to the spin–orbit splitting of the conduction band, spin-singlet and spin-triplet interlayer excitons with opposite helicities in the same valley configuration and atomic registry were both theoretically predicted^134^ and experimentally identified^110,135^ in TMD vdW heterostructures, suggesting very rich but also complicated optical properties for the interlayer excitons.

Note that the magnetic dipole of the interlayer exciton is also quite different from that of the intralayer exciton, as manifested by their distinct *ɡ* factors. An effective *ɡ* factor of −15 was reported for the interlayer excitons in a MoSe_2_/WSe_2_ heterostructure with a twist angle of ~54°, which is much larger than that of intralayer excitons in TMD monolayers (approximately −4 in most cases^138–140^), thus producing giant valley Zeeman splitting of the interlayer excitons with near-unity valley polarization under magnetic fields^25^. A recent study also demonstrated large *ɡ* factors for both spin-singlet (∼10.7) and spin-triplet (∼15.2) interlayer excitons in a MoSe_2_/WSe_2_ heterostructure with a 60° twist angle^110^. Moreover, the *ɡ* factor of the interlayer excitons was found to be stacking-dependent. For instance, the *ɡ* factors of interlayer excitons in MoSe_2_/WSe_2_ heterostructures with twist angles of 2° and 57° were determined to be approximately 6.7 and −15.9, respectively^141^. Intriguingly, the *ɡ* factor of interlayer excitons in MoSe_2_/WSe_2_ heterostructures with a twist angle approaching 60° (H-type configuration) was always larger than that with a twist angle approaching 0° (R-type configuration)^25,110,117,141,142^, and the corresponding sign was opposite for the two stacking configurations^141^. These experimental findings were further revealed in a recent theoretical study^143^. Larger effective *ɡ* factors with opposite signs were theoretically predicted for the interlayer excitons in the H-type (60°) MoSe_2_/WSe_2_ heterostructure^143^ than for those in the R-type (0°) system, which matched the experimental results well. The differences of *ɡ* values and their signs between the R-type and H-type heterostructures were interpreted as distinct valley pairings for the conduction and valence bands in the two systems^141,143^. In the H-type system, the valley indexes for the conduction and valence bands were opposite ((+K, −K′) or (−K, +K′)), while those in the R-type system were the same ((+K, +K′) or (−K, −K′)). This consequently produced a larger effective *ɡ* factor in the H-type system (but with the opposite sign), when the *g* factor of the conduction band minus that of the valence band^141,143^. In addition, the theoretical analysis showed that the spin-flip transitions could provide an extra spin contribution to the *ɡ* factor, leading to the effective *ɡ* factor for the spin-triplet interlayer excitons being higher than that for spin-singlet interlayer excitons^143^, which is consistent with the above experimental results^110^. These studies demonstrate that the *ɡ* factor of interlayer excitons in TMD vdW heterostructures is both spin-dependent and stacking-dependent, and most importantly, that the measured *ɡ* values and their signs can serve as a valuable basis for determining the nature of the interlayer excitons (such as spin-singlet or spin-triplet and R-type or H-type).

### Moiré interlayer excitons

**Fig. 4:**
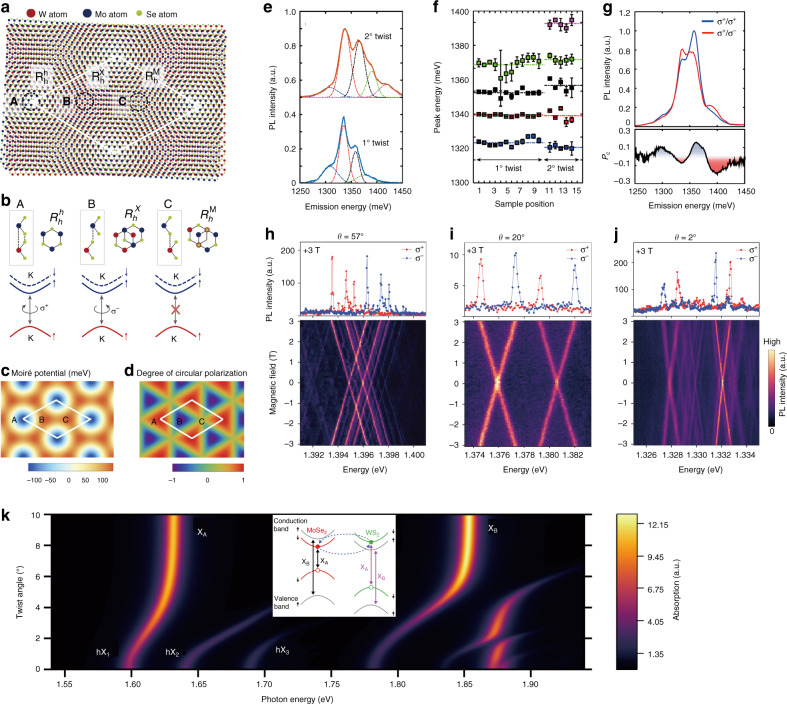
Moiré interlayer exciton. **a** Moiré pattern in an R-type MoSe_2_/WSe_2_ heterobilayer^150^. The three highlighted regions (A, B, and C sites) correspond to the local atomic configurations with three-fold rotational symmetry. **b** Side-views and top-views of the three R-type local atomic registries (A, B, and C sites) and the corresponding optical selection rules for interlayer excitons in these atomic registries^150^. The interlayer exciton emission at the A (B) site is left-circularly (right-circularly) polarized, while that at the C site is transition-forbidden under normal incidence. **c** Moiré potential of the interlayer exciton transition with a local minimum at site A^150^. **d** Optical selection rules for K-valley interlayer excitons^150^. The high-symmetry A and B sites are circularly polarized with opposite signs, and the regions in between are elliptically polarized. **e** PL spectra of multiple moiré interlayer excitons in MoSe_2_/WSe_2_ heterobilayers with twist angles of 1° (bottom) and 2° (top). Each spectrum is fitted with four (1°) or five (2°) Gaussian functions^150^. **f** The center energy of each moiré interlayer exciton resonance at different spatial positions across each sample^150^. The average peak spacing for a twist angle of 1° (2°) is 22 ± 2 meV (27 ± 3 meV). **g** Circularly polarized PL spectrum of the 1° sample under σ+ excitation (top)^150^. The degree of circular polarization versus the emission wavelength is shown at the bottom, demonstrating multiple moiré interlayer excitons with alternating co-circularly and cross-circularly polarized emissions. **h**–**j** Magnetic-field-dependent PL from moiré-trapped interlayer excitons in MoSe_2_/WSe_2_ heterobilayers with twist angles of 57° (**h**), 20° (**i**), and 2° (**j**)^141^. Top: Circular polarization-resolved PL spectra with narrow linewidth (100 μeV) at 3 T. The excitation is linearly polarized, and the σ^+^ and σ^−^ components of the PL emission are shown in red and blue, respectively. Bottom: total PL intensity as a function of magnetic field, displaying a linear Zeeman shift of the σ^+^-polarized and σ^−^-polarized components. The derived effective *g*-factors from Zeeman splitting are −15.89 ± 0.02, −15.79 ± 0.05, and 6.72 ± 0.02 for samples with twist angles of 57°, 20°, and 2°, respectively. **k** Absorption spectrum of the MoSe_2_/WS_2_ heterobilayer as a function of the twist angle^155^. The MoSe_2_ A-exciton and B-exciton resonances (*X*_A_ and *X*_B_) are indicated for large twist angles where hybridization effects become negligible. The three resonances labeled hX_1,2,3_ appearing at *θ* ≈ 0° correspond to the hybridized excitons in the vicinity of *X*_A_. Those in the vicinity of *X*_B_ are not labeled. Specifically, hX_3_ results from the hybridization of the first folding of the *X*_A_ band into the mini Brillouin zone (the reduced BZ of the moiré superlattice), a direct signature of the moiré superlattice effect. **a**–**g** Reprinted with permission from ref. ^150^ [Springer Nature Limited]. **h**–**j** Reprinted with permission from ref. ^141^ [Springer Nature Limited]. **k** Reprinted with permission from ref. ^155^ [Springer Nature Limited]

Intrigued by the amazing discovery of Mott insulating states and unconventional superconductivity in magic-angle twisted graphenes^144,145^, significant interest has been ignited in twisted 2D vdW heterobilayers featuring moiré patterns or superlattices^18,36,38,41,47,116,134,141,146–156^, which has greatly flourished in the new research area called “twistronics”. For TMD vdW heterobilayers with a small lattice mismatch and/or rotational misalignment, a moiré pattern could be formed naturally with periodic changes in the interlayer atomic registry, and the corresponding moiré period varying from several nanometers to tens of nanometers is given by $b \approx a/\sqrt {\delta ^2 + \theta ^2}$, where *a* is the monolayer lattice constant, *δ* is the lattice mismatch, and *θ* is the relative twist angle^36,116^. In such a moiré superlattice, the local interlayer atomic registry varies continuously with three high-symmetry sites (A, B, and C) preserving the three-fold rotational symmetry $\hat C_3$ (Fig. 4a, b)^150^, which are typically denoted by ($R_{\rm{h}}^{\rm{h}}$, $R_{\rm{h}}^{\rm{X}}$, $R_{\rm{h}}^{\rm{M}}$) or ($H_{\rm{h}}^{\rm{h}}$, $H_{\rm{h}}^{\rm{X}}$, $H_{\rm{h}}^{\rm{M}}$) (*X*: chalcogen site, *M*: metal site, and *h*: hollow center of the hexagon) for R-type or H-type stacked heterobilayers. $R_{\rm{h}}^{\rm{u}}$ ($H_{\rm{h}}^{\rm{u}}$, *u* = *X*, *M*, *h*) means that the *u* site of the electron layer overlaps vertically with the *h* site of the hole layer. As reported, this alteration of the local interlayer atomic registry across a moiré superlattice could consequently lead to lateral modulation of both the interlayer distance and the local band gap, producing a position-dependent potential (i.e., moiré potential) for confining/trapping excitons with a potential modulation depth of ~100–250 meV, and the local potential minima sitting at those high-symmetry sites within the superlattice (Fig. 4c)^18,36,148–151^.

Obviously, for interlayer excitons with electrons and holes separated in opposite layers, their properties should be substantially affected by the interlayer configurations and thus by the moiré pattern, which carries periodically varied interlayer atomic registries. As proposed, an intuitive impact of the moiré pattern on interlayer excitons is that it is able to trap them in the minima of the moiré potential, and thereby, an ordered nanoscale quantum emitter array or an excitonic superlattice could be realized with interlayer excitons by designing an appropriate moiré period and/or the profile of the moiré potential, which can be tuned via an external electric field or strain^36,69^. Another intriguing effect from the moiré pattern is the lateral modulation of the optical selection rules within the superlattice^36,150^. That is, an interlayer exciton located at the high-symmetry site $R_{\rm{h}}^{\rm{h}}$ ($R_{\rm{h}}^{\rm{X}}$) couples only to *σ*^+^ (*σ*^−^) circularly polarized light for a specific valley and at sites in between is elliptically polarized, while an interlayer exciton located at site $R_{\rm{h}}^{\rm{M}}$ couples to out-of-plane polarization light (Fig. 4b, d)^150^. This means that even interlayer excitons in the same spin-valley configuration can couple to polarized light with opposite helicities due to the preserved $\hat C_3$ symmetry at these high-symmetry sites, suggesting that circular polarization selection rules are no longer locked to the valley index. In other words, the optical selection rules in such heterobilayers are determined not only by the atomic quantum number (associated with the spin, orbital, and valley quasi-particle angular momenta (QAM)), but also by the interlayer translation that characterizes the local interlayer atomic registry in the moiré superlattice (referring to moiré QAM)^152^. Therefore, a new degree of freedom, i.e., the moiré degree of freedom, is introduced to better characterize and manipulate the physical properties of interlayer excitons in the moiré pattern. The optical dipole oscillator strength and radiative lifetime of interlayer excitons were reported to be modulated on a few orders of magnitude across the superlattice by such a moiré effect^148^. Moreover, the emerging periodicity from the strong-coupling moiré superlattices could result in folding of the interlayer (intralayer) exciton bands into a mini-Brillouin zone (a reduced BZ of the moiré superlattice), forming moiré exciton minibands manifested as multiple exciton resonances with different optical selection rules in the optical spectra^36,41,116,146^.

Note that the discussed intriguing phenomena realized by the moiré effect have so far been explored mainly by theoretical calculations in twisted TMD vdW heterobilayers. Recently, multiple moiré interlayer exciton resonances with alternating co-circularly and cross-circularly polarized emission and almost a constant peak spacing (~22–27 meV) were experimentally observed in a MoSe_2_/WSe_2_ heterobilayer by far-field optical measurements at 15 K (Fig. 4e–g)^150^. These resonances were attributed to the excitonic ground and excited states confined within the moiré potential with a depth of ~100 meV, consistent with theoretical calculations. Subsequently, multiple interlayer exciton states with opposite optical selection rules were reported in a WSe_2_/WS_2_ moiré superlattice, and their spin, valley and moiré QAM, which contribute to the optical selection rules, were unambiguously determined via novel resonant optical pump-probe spectroscopy and PL excitation spectroscopy^152^. Moiré-trapped interlayer excitons with narrow linewidth (100 μeV), circularly polarized optical selection rules and large Landé *g*-factor at various twist angles were also evidenced in MoSe_2_/WSe_2_ heterobilayers by both optical and magneto-PL spectroscopy under very low excitation power (20 nW) and temperature (1.6 K) (Fig. 4h–j)^141^. Such moiré-trapped interlayer excitons with characterized narrow linewidths were recently reported in a trilayer heterostructure with a WSe_2_ monolayer below the MoSe_2_ bilayer, where the resulting two quantum-confined interlayer excitons possessed distinct spin-layer-valley configurations (with parallel and antiparallel spin-valley-locked magnetic moments)^153^. Although intralayer excitons are less influenced by the moiré effect, when the moiré potential is sufficiently strong, such as up to ~250 meV, multiple moiré intralayer exciton resonances can be experimentally detected, as revealed in WSe_2_/WS_2_ heterostructure superlattices^151^. More intriguingly, hybridization of the moiré intra- and interlayer exciton minibands could even occur in the moiré mini-Brillouin zone when the CB edges of the constituent monolayers are closely aligned, leading to hybridized excitons that inherit both the brightness of intralayer excitons and the polar nature of interlayer excitons, accompanied by a pronounced twist-angle-controlled energy shift (Fig. 4k)^155^. Such a hybridization effect was also experimentally demonstrated in homobilayers, along with the observation of an incompressible Mott-like state of electrons at half-filling of each layer^47,154^. Additionally, hybridization between the moiré interlayer excitons and photons in a planar 2D cavity could be possible and result in two types of moiré polaritons with distinct forms of topological transport phenomena such as spin/valley Hall and polarization Hall effects^156^. In summary, moiré superlattices provide a rich playground for exploring new quantum phenomena in vdW heterostructures, which undoubtedly will facilitate their application in various exciting areas such as nanophotonics and quantum information processing.

## Interlayer exciton relaxation in TMD vdW heterostructures

In this section, we review the population recombination dynamics of interlayer excitons, the intervalley scattering process, and the valley polarization dynamics of interlayer excitons in TMD vdW heterostructures.

### Recombination dynamics of interlayer excitons

Owing to the reduced overlap of the electron and hole wavefunctions of the spatially indirect interlayer exciton, its oscillation strength was reported to be several orders of magnitude lower than that of the intralayer exciton, as stated before^80,116^, which indicates a much longer recombination lifetime, as evidenced in both theoretical and experimental studies^22–28,33,42,56,111,157–161^.

**Fig. 5:**
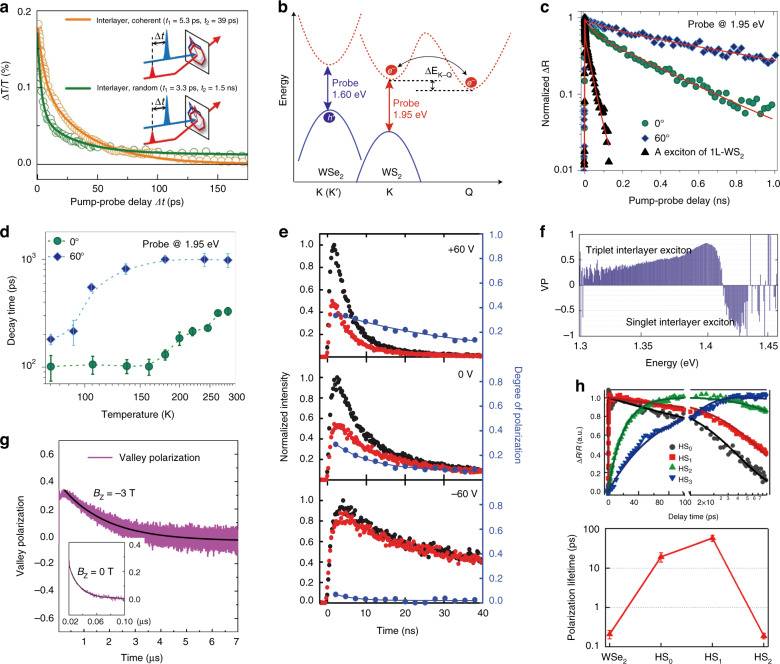
Population recombination and valley polarization dynamics of interlayer excitons. **a** Transient dynamics of interlayer excitons at 1.6 eV in coherently stacked and randomly stacked MoS_2_/WS_2_ heterobilayers^42^. **b** Schematic illustration of probing the electron (K valley of WS_2_ at 1.95 eV) and hole (K valley of WSe_2_ at 1.60 eV) dynamics in a WS_2_/WSe_2_ heterostructure using a pump energy of 1.58 eV^91^. The hole population relates to the sum of the K–K and K–Q exciton populations, while the electron population reflects only the K–K exciton population. Δ*E*_K–Q_ represents the energy difference between the lowest-energy K–Q and K–K transitions. **c** Electron dynamics of WS_2_/WSe_2_ heterostructures with twist angles of 0° and 60° at 295 K^91^. The exciton dynamics of monolayer WS_2_ are also shown. **d** Electron dynamics as a function of temperature for WS_2_/WSe_2_ heterostructures with twist angles of 0° and 60°^91^. **e** Co-polarized (σ^+^, black curves) and cross-polarized (σ^−^, red curves) PL dynamics of interlayer excitons under σ^+^ excitation at selected gate voltages^33^. The valley polarization dynamics are shown in blue curves. **f** Valley polarization (VP) of the singlet and triplet interlayer excitons with opposite helicities in the MoSe_2_/WSe_2_ heterobilayer^135^. **g** Valley polarization dynamics of interlayer excitons in the MoSe_2_/WSe_2_ heterobilayer extending to ~μs time scale under an out-of-plane magnetic field (B_z_)^27^. **h** Top: the interlayer exciton recombination kinetics for WS_2_/WSe_2_ heterostructures with 0, 1, 2, and 3 hBN intermediate layers, denoted HS_0_, HS_1_, HS_2_, and HS_3_, respectively; Bottom: the valley polarization lifetime in the WSe_2_ monolayer, HS_0_, HS_1_, and HS_2_^160^. **a** Reprinted with permission from ref. ^42^ [Macmillan Publishers Limited]. **b**–**d** Reprinted with permission from ref. ^91^ [Springer Nature Limited]. **e** Reprinted with permission from ref. ^33^ [American Association for the Advancement of Science]. **f** Reprinted with permission from ref. ^135^ [American Physical Society]. **g** Reprinted with permission from ref. ^27^ [Springer Nature Limited]. **h** Reprinted with permission from ref. ^160^ [American Chemical Society]

Ultrafast optical measurements such as femtosecond pump-probe and time-resolved PL (TRPL) spectroscopy are typical tools for tracing the recombination dynamics of interlayer excitons. An early report demonstrated direct probing of the transient spectrum and dynamics of interlayer excitons in MoS_2_/WS_2_ heterobilayers by an ultrafast pump-probe technique, with its recombination being tuneable from direct (~40 ps) to indirect (~1.5 ns) dependent on the stacking orientation (Fig. 5a)^42^. With this technique, the long lifetime property of interlayer excitons could be indirectly reflected by the photobleaching signals of the intralayer excitons in heterobilayers being longer than those in monolayers^56,159,160^. Nevertheless, due to the very small oscillation strength imposing difficulty on directly accessing the absorption transition, PL dynamics are most commonly used to directly reflect the interlayer exciton recombination process, with the reported PL lifetimes ranging from several ns to hundreds of ns or even ~μs^22–28,33,111,161^. Additionally, a number of studies have revealed that the recombination lifetimes of interlayer excitons can be extensively tuned by external factors, such as the electric field^26,33^, temperature^23,24,28,161^, twisting angle^56^, interlayer distance^160^, and optical cavity^111^ due to their interlayer configuration (or spatially indirect nature). Note that double interlayer excitons with two distinctly long-lived lifetimes were observed in certain TMD vdW heterostructures, with their assignments as momentum direct and indirect interlayer excitons^23^, spin-singlet and spin-triplet interlayer excitons^22,135^, or neutral and charged interlayer excitons^161^. The origin of these two species and their accurate assignments are of fundamental interest for further exploration.

The decay lifetimes of interlayer excitons in various TMD vdW heterostructures are summarized in Table 1. The interlayer exciton lifetime of MoSe_2_/WSe_2_ heterostructures has been most widely studied and generally consists of two lifetime components: a fast component in tens of ns and a slow component in hundreds of ns. Some results reported only one of these two lifetimes, either in several ns or in hundreds of ns (Table 1). The accurate assignment of these two lifetimes remains ambiguous, although they were previously ascribed to quasi-direct (fast) and indirect (slow) interlayer excitons^23^. Additionally, longer lifetimes were usually observed at lower temperatures, probably due to suppressed non-radiative recombination. Note that a dark exciton in the MoSe_2_/WSe_2_ heterostructure reportedly had a lifetime on a microsecond timescale, and could serve as a microsecond reservoir for an interlayer exciton^27^, which is quite promising for the realization of excitonic devices with long exciton transport distances. The interlayer exciton lifetime in WS_2_/WSe_2_ heterostructures was reported to be relatively short, with a value within 1 ns, which could be elongated to several ns or longer than 10 ns by either lowering the temperature or increasing the interlayer distance between the constituent monolayers by inserting hBN layers (Table 1). The MoS_2_/WS_2_ heterostructure was also reported to have a relatively short interlayer exciton lifetime, with values on the order of tens of ps or on the order of ns depending on the stacking mode between layers^42^. Such a stacking mode or twist angle dependence was also revealed in the MoS_2_/WSe_2_ heterostructure, with lifetimes ranging from ~50 ps to ~3 ns^56^. Surprisingly, the interlayer exciton lifetime in the hBN-capsulated MoS_2_/WS_2_ heterostructure was found to be significantly longer (~100 ns)^26^ than that of the above uncapsulated MoS_2_/WS_2_ heterostructure^42^, the reason for which is obscure and may be related to complicated factors such as the different material structures, fabrication methods, temperatures, twist angles between layers, and detection techniques.

**Table 1:** Decay lifetimes of interlayer excitons in various TMD vdW heterostructures

HSs	Fabrication method	Twist angle (°)	Lifetime (ns)			Temp (K)	Technique	Notes	Refs.
			*τ*_1_	*τ*_2_	*τ*_3_				
MoSe_2_/WSe_2_	MEwet transfer		1.80			20	TRPL		^22^
	MEdry transfer	0	~10			30	TRPL		^33^
	MEdry transfer		16	138		4.5	TRPL		^28^
	MEdry transfer	54	40	>100		4	TRPL	*B* = 0 T	^25^
		54	70	>200		4	TRPL	*B* = 28 T	
	MEdry transfer	0/60	10	100		4	TRPL	*E*_HS_ = 0 V/nm	^30^
		0/60		600		4	TRPL	*E*_HS_ > 0.1 V/nm	
	MEdry transfer	60	~50	~100		3	TRPL		^23^
	MEdry transfer	0/60		~1000			TRPL	Dark exciton	^27^
	MEdry transfer	58.7	2.30				TRPL		^135^
	MEdry transfer	60	~12			4	TRPL	Moiré interlayer exciton	^142^
	MEdry transfer	3.5		100			TRPL	Moiré period (5 nm)	^162^
	MEdry transfer	1.1	1.00				TRPL	Moiré period (17 nm)	
	CVD	0	1.20	5.50			TRPL	No moiré pattern	
	CVD	60	6.00	44	877	4.2	TRPL	No moiré pattern	^111^
WS_2_/WSe_2_	CVD	0, 60	0.97			295	Pump-probe	Hole dynamics (WSe_2_)	^91^
		0, 60	4.10			78	Pump-probe	Hole dynamics (WSe_2_)	
WS_2_/WSe_2_	CVD	0	0.36			295	Pump-probe	Electron dynamics (WS_2_)	^91^
		60	0.98			295	Pump-probe	Electron dynamics (WS_2_)	
WS_2_/WSe_2_	MEdry transfer		0.41				Pump-probe	Electron dynamics (WS_2_)	^160^
WS_2_/hBN/WSe_2_	MEdry transfer		1.00				Pump-probe	Electron dynamics (WS_2_)	^160^
WS_2_/bilayer-hBN/WSe_2_	MEdry transfer		4.96				Pump-probe	Electron dynamics (WS_2_)	^160^
WS_2_/trilayer-hBN/WSe_2_	MEdry transfer		>10				Pump-probe	Electron dynamics (WS_2_)	^160^
MoS_2_/WS_2_	Vertical heteroepitaxial growth	Coherent stack	0.04				Pump-probe		^42^
	Manual stacking	Random stack	1.50				Pump-probe		
hBN/MoS_2_/WS_2_/hBN	MEdry transfer	0/60		~100		10	TRPL	*V*_G_ = 0–4.5 V	^26^
				~400		10	TRPL	*V*_G_ = −4.5 V	
MoS_2_/WSe_2_	MEdry/wet transfer		~0.05 to ~3				Pump-probe	Depending on the twist angle	^56^
MoS_2_/MoSe_2_/MoS_2_	CVDwet transfer		5	135		6	TRPL		^24^
WSe_2_/MoSe_2_/WSe_2_	MEdry transfer		2.54			4	TRPL	Neutral interlayer exciton	^161^
			2.47			40			
			1.84			80			
			0.41			120			
WSe_2_/MoSe_2_/WSe_2_	MEdry transfer		1.24			4	TRPL	Charged interlayer exciton	^161^
			0.47			20			

*HSs* heterostructures, *Temp* temperature, *ME* mechanical exfoliation, *CVD* chemical vapor deposition, *TRPL* time-resolved photoluminescence spectroscopy, *B* applied out-of-plane magnetic field, *E*_HS_ electric field across the heterostructure, *VG* externally applied voltage, *Ref* reference

Additionally, the interlayer exciton lifetimes obtained by the pump-probe technique were generally shorter than those detected by TRPL spectroscopy for the heterobilayers (Table 1). Due to the small oscillation strength of the interlayer excitons, the pump-probe technique normally detected the electron/hole dynamics of the constituent monolayers to reflect the interlayer exciton lifetime, while TRPL directly detected the PL dynamics of the interlayer excitons. In addition, the pump-probe method was generally performed at room temperature or at higher temperatures in the currently reported works (for those specified in the works). Both of these factors may affect the detected interlayer exciton lifetimes. Nevertheless, the clear origin of these discrepancies needs further investigation. Interestingly, interlayer excitons were also found in vdW heterostructures with three constituent monolayers. For instance, a long-lived interlayer exciton emission was reported in the MoS_2_/MoSe_2_/MoS_2_ heterostructure with two lifetime components (~5 and ~135 ns, ascribed to momentum-direct and momentum-indirect interlayer excitons)^24^. In another trilayer heterostructure, WSe_2_/MoSe_2_/WSe_2_^161^, both neutral and charged interlayer excitons were spectrally resolved with lifetimes on the order of ns, which decreased linearly as the temperature increased, most likely due to enhanced non-radiative recombination at higher temperatures. Compared to the bilayer MoSe_2_/WSe_2_, the interlayer excitons formed in the trilayer WSe_2_/MoSe_2_/WSe_2_ were demonstrated to have greater electron-hole wavefunction overlap and thus stronger oscillation strength, which may account for the observed lifetime discrepancies between these two types of heterostructures. Lifetime modulation of the interlayer exciton by changing its electron-hole wavefunction overlap was also realized by applying an external electric field^26,30^, or by tuning the interlayer distance as described above^160^, with a longer interlayer exciton lifetime observed with reducing electron-hole wavefunction overlap.

Moreover, the role of the moiré superlattice on the interlayer exciton lifetime was discussed recently. A twist angle dependence of the interlayer exciton lifetime was demonstrated in the WS_2_/WSe_2_ heterostructure, which exhibited a deeper moiré potential for a twist angle of 0° than a twist angle of 60° (Fig. 5b–d)^91^. Figure 5b shows a schematic illustration of probing the electron and hole dynamics in the WS_2_/WSe_2_ heterostructure to reflect the interlayer exciton dynamics by using the pump-probe technique with a pump energy of 1.58 eV. As stated in this work, the hole population (in the K valley of WSe_2_ at 1.60 eV) monitored the sum of the K–K and K–Q interlayer exciton populations, while the electron population (in the K valley of WS_2_ at 1.95 eV) reflected only the K–K exciton population. As shown in Fig. 5c, the dynamics probed at 1.95 eV (related to the K–K interlayer exciton) exhibited a substantial twist angle dependence, with the decay lifetime for 60° being three times longer than that for 0° at 295 K. Both lifetimes decreased as the temperature decreased (Fig. 5d). Such twist angle-dependent and temperature-dependent dynamics were explained by the intraband scattering of the electrons between the K and Q valleys. Since the moiré potential for 0° was deeper or larger than that for 60°, as was the energy difference between the lowest-energy K–Q and K–K transitions (Δ*E*_K–Q_)^91^, the larger Δ*E*_K–Q_ for 0° resulted in a less efficient back-scattering of electrons from Q to K and thus a shorter K–K exciton decay lifetime than those for 60°. This was consistent with the phenomenon of the temperature dependence of the lifetimes. At higher temperatures, more electrons could be back-scattered from the Q to K valley by phonon absorption, which could increase the K–K exciton population and hence give rise to a longer decay lifetime (Fig. 5d). Note that a higher temperature was required to back-scatter electrons for 0° than for 60° due to the larger Δ*E*_K–Q_ for 0° (Fig. 5d). The moiré effect on the interlayer exciton lifetime was also studied in MoSe_2_/WSe_2_ heterostructures prepared by chemical vapor deposition (CVD, twist angle of 0°) and mechanical stacking with twist angles of ~1.1° and ~3.5°^162^. The sample at a twist angle of 0° without a moiré pattern possessed an averaged interlayer exciton lifetime of ~1.7 ns, which was similar to that at a twist angle of ~1.1° with a moiré period of ~17 nm. However, the interlayer exciton lifetime in the sample with a twist angle of ~3.5° and a moiré period of ~5 nm was significantly longer (~100 ns), which was ascribed to the larger momentum mismatch between the CBM and VBM at this twist angle, thus inducing an indirect transition in both real and momentum space.

To date, the interlayer exciton lifetimes studied in various TMD vdW heterostructures have been discussed, with the reported values covering a broad range from nanoseconds to microseconds. Many factors can influence the interlayer exciton lifetime such as the temperature, the degree of electron-hole overlap, and the moiré superlattice. Generally, longer lifetimes were observed at lower temperatures, probably due to suppressed non-radiative recombination, except for the case with the moiré effect^91^. The electron-hole wavefunction overlap of the interlayer exciton should be the main source for modulating its lifetime, and it could be influenced by various factors such as the material structure, fabrication method, twist angle, and external electric field, giving rise to a longer lifetime under a smaller electron-hole overlap. Although more exploration is needed to clarify the discrepancies in these studies, the observed longer recombination lifetimes of interlayer excitons compared to those of intralayer excitons, plus the existing repulsive interactions, make interlayer excitons quite suitable for exploitation in excitonic devices with long-distance exciton transport and promising for macroscopic quantum state generation for the realization of Bose–Einstein condensation and superfluidity.

### Intervalley scattering process

Intervalley scattering plays a key role in determining the valley polarization lifetimes of materials for valleytronic applications. Due to the valley-dependent optical selection rules and the spin-valley locking effect, the intervalley scattering in TMD monolayers was expected to be slow due to the large momentum mismatch along with a simultaneous spin flip between the +K and −K valleys^3,133^. However, this was in contrast to the experimental observations, with the reported spin–valley lifetimes on a time scale of a few picoseconds in TMD monolayers even at low temperatures^163–166^. Such contradictory findings were attributed to the strong electron-hole exchange interactions (via the Maialle-Silva-Sham mechanism) for the intralayer excitons in monolayers^167–169^, which significantly strengthen the intervalley scattering process and thus shorten the spin–valley lifetimes. Undoubtedly, this will impede the application of TMDs in spintronics/valleytronics if intralayer excitons are used as spin–valley information carriers.

On the other hand, the interlayer exciton with transferred spin–valley polarization has been considered a good candidate for exploring valleytronics. Since the electron-hole exchange strength is proportional to the oscillation strength^168^, such an exchange interaction could be dramatically weakened for the interlayer exciton due to its oscillation strength being much smaller than that of the intralayer exciton. Therefore, the intervalley scattering process of the interlayer exciton should be strongly suppressed^167^, giving rise to significantly extended valley lifetimes, as discussed below.

### Valley polarization dynamics of interlayer excitons

Owing to their intrinsic valley-contrasting physics and spin–valley coupling properties^132,133^, TMDs offer an appealing platform for developing spintronic/valleytronic devices by using spin–valley pseudospin, which can potentially circumvent the limitations of speed and power consumption imposed by electron charges. A sufficiently long spin–valley lifetime is essential for such applications to maintain the spin–valley information for subsequent signal processing or communication.

Interlayer excitons, which have a long population lifetime and suppressed intervalley scattering, as discussed above, have been considered promising spin–valley information carriers due to their valley polarization lifetime being longer than that of intralayer excitons. An early study demonstrated ~30% valley-polarized interlayer exciton emission at 30 K in a MoSe_2_/WSe_2_ heterobilayer, with a remarkably enhanced valley polarization lifetime of a few nanoseconds (compared to the few picosecond valley lifetime of intralayer excitons)^33^. The degree of valley polarization could be further electrically controlled by the gate, with reported valley polarization lifetimes of ~39, ~10, and ~5 ns for gate voltages of +60, 0, and −60 V, respectively (Fig. 5e)^33^. This phenomenon was most likely due to the larger interlayer separation of the electron and hole at positive gate voltages; thus, a smaller oscillation strength and weaker intervalley scattering resulted in a higher valley polarization and longer valley polarization lifetime. However, obtaining a solid microscopic mechanism responsible for the observed gate-dependent valley polarization lifetimes requires thorough future investigations. A much higher degree of valley polarization approaching unity or over 80% with a valley polarization lifetime on tens of nanoseconds was later achieved for both singlet and triplet interlayer excitons in MoSe_2_/WSe_2_ heterobilayers encapsulated with hexagon-boron nitride (hBN) layers at cryogenic temperatures (Fig. 5f)^135^. This high valley polarization was generally ascribed to the ultrafast spin-valley conserved charge transfer as well as the suppressed intervalley scattering of the interlayer excitons. Alternatively, valley polarization could be enhanced by applying an out-of-plane magnetic field (**B**_z_) to lift the valley degeneracy via the valley-Zeeman effect^25,27,110^. Giant valley-Zeeman splitting (~26 meV) of the interlayer excitons with near-unity valley polarization and a large effective *g*-factor (−15) was observed in AB-stacked (stacking angle close to 60°) MoSe_2_/WSe_2_ heterobilayers under a 30 T magnetic field^25^. As expected, the valley polarization lifetime was extended under the magnetic field, with the reported value increasing from ~15 ns (**B**_z_ = 0 T) to ~1.75 μs at **B**_z_ = −3 T (Fig. 5g)^27^. In addition to the electric and magnetic field control of the valley polarization and its dynamics, structural tuning by inserting hBN intermediate layers between the bilayer to modulate the interlayer electron-hole Coulomb interaction was recently demonstrated to precisely control the interlayer exciton recombination and the valley polarization dynamics (Fig. 5h)^160^. Notably, a single particle such as the resident hole in the heterobilayer emerged as a good valley information carrier. This single particle could have a much longer valley polarization lifetime (>20^170^ or 40 μs^171^ at low temperature) than the interlayer exciton owing to the lack of electron-hole exchange interaction and the large momentum mismatch, and the spin–orbit coupling resulted in a high energy barrier in the VBs between the *K* valleys, all of which could dramatically suppress the intervalley scattering and prolong the recombination lifetime to substantially extend the valley polarization lifetime.

According to the reported results, it is obvious that the valley polarization and the dynamics of the interlayer exciton basically rely on three factors: (1) the initial degree of valley polarization inherited from the intralayer exciton, (2) the interlayer exciton recombination lifetime, and (3) the intervalley scattering of the interlayer exciton. The first factor is tightly linked to the interlayer spin–valley conserved charge transfer and the intralayer recombination, as well as the intralayer valley depolarization process. Higher valley polarization inherited by the interlayer exciton is expected if interlayer charge transfer occurs at timescales much faster than both intralayer recombination and valley depolarization. The second influencing factor is straightforward since no valley-polarized interlayer excitons exist when their population decays to zero. The final factor is closely related to the electron-hole exchange interaction, as stated above. All these factors ought to be strongly affected by the interlayer coupling in the heterostructures, and additional efforts to engineer these factors to further extend the valley polarization lifetimes of the interlayer excitons are expected.

## Interlayer exciton transport in TMD vdW heterostructures

In this section, we review the interlayer exciton diffusion and drift, valley-polarized interlayer exciton transport, and interlayer exciton transport under moiré potential in TMD vdW heterostructures.

### Interlayer exciton diffusion and drift

Solid-state devices based on the manipulation of excitons hold great potential for bridging the optical communication and signal processing modules in integrated circuits^172,173^. Controllable manipulation of the exciton transport in these devices requires that excitons possess a sufficiently long lifetime to travel over large distances, and that their energy is sensitive to the applied electric field^172,173^. Apparently, the conventional exciton in direct-gap semiconductors has difficulty meeting such requirements due to its short recombination lifetime (<1 ns) and the absence of a built-in dipole moment^174,175^. However, a spatially indirect exciton, with a permanent dipole moment and a lifetime several orders of magnitude longer than that of conventional excitons, is a good candidate for these excitonic devices. In fact, indirect excitons formed in traditional semiconductor coupled quantum wells (CQWs) have been extensively explored, with observed lifetimes varying from nanoseconds to microseconds and transport distances over tens and hundreds of micrometers within the lifetimes^176–185^. On the other hand, the emerged TMD vdW heterostructures provide a new platform for generating indirect excitons with long recombination lifetimes, as discussed above, which has recently ignited intensive interest in studying exciton transport in these systems for potential novel excitonic devices.

**Fig. 6:**
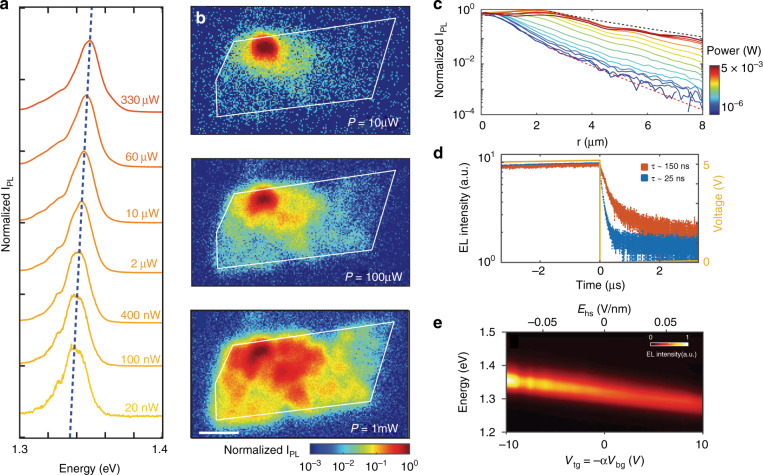
Interlayer exciton diffusion. **a** Power dependence of the normalized PL spectra of interlayer excitons in a MoSe_2_/WSe_2_ heterobilayer^30^. **b** Spatial dependence of the normalized PL intensity of interlayer excitons for different incident powers (10, 100, and 1000 μW) at 4 K^30^. The white outlines show the MoSe_2_/WSe_2_ heterostructure area. The laser excitation spot is fixed at the top left of the sample. Scale bar, 5 μm. **c** Normalized PL intensity of interlayer excitons versus distance from the excitation point under different excitation powers^30^. **d** Time-dependent electroluminescence (EL) intensity of the neutral (orange, ~150 ns) and charged (blue, ~25 ns) interlayer excitons in the MoSe_2_/WSe_2_ heterobilayer^30^. **e** EL energy of the interlayer exciton versus the electric field (*E*_hs_) applied vertically to the MoSe_2_/WSe_2_ heterostructure^30^. **a**–**e** Reprinted with permission from ref. ^30^ [American Association for the Advancement of Science]

For instance, interlayer exciton diffusion in various TMD vdW heterostructures (i.e., a MoSe_2_/hBN/WSe_2_ heterotrilayer and MoSe_2_/WSe_2_ heterobilayer at cryogenic temperature (4 K)) were investigated recently^30,34^. Both the PL emission energy and the diffusion distance increased as the laser power increased (Fig. 6a–c)^30^, which was principally ascribed to density-dependent exciton–exciton repulsion. At the applied highest laser power, the interlayer excitons could diffuse several micrometers away from the excitation spot^34^ or even across the whole sample^30^. Additionally, when free charge carriers were introduced by electrostatically doping one of the TMD layers, charged interlayer excitons that could be controlled by an in-plane electric field were observed, and they could also drift across the entire heterostructure (MoSe_2_/WSe_2_) under an applied bias voltage^30^. More intriguingly, in this MoSe_2_/WSe_2_ heterobilayer, the electrical generation of both long-lived neutral (~150 ns) and charged (~25 ns) interlayer excitons was realized by free-carrier injection using ohmic contacts in individual monolayers, and the electroluminescence (EL) energy of the interlayer excitons could be tuned in ranges of over hundreds of meV with the external electric field (Fig. 6d, e)^30^, similar to the behavior of PL emission due to the Stark effect. This achievement is meaningful since an electrically driven near-infrared light source, in addition to signal processing devices relying on transport characteristics, is highly desirable in optoelectronic integrated circuits.

**Fig. 7:**
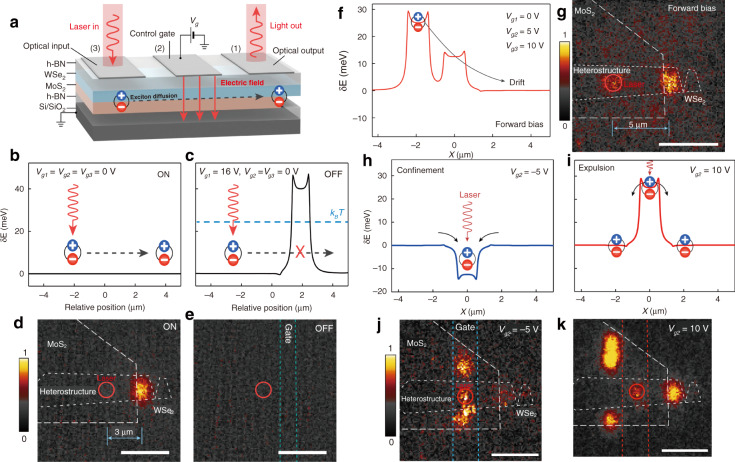
Control of the interlayer exciton transport by a laterally modulated potential landscape at room temperature. **a** Schematic illustration of the MoS_2_/WSe_2_ heterostructure encapsulated in hexagonal boron nitride (h-BN) with top and bottom gates^29^. The three gate voltages (*V*_g1_, *V*_g2_, and V_g3_) applied to the transparent graphene electrodes (gates 1–3) can be engineered to provide a potential landscape for controlling the interlayer exciton transport through the device. **b**, **c** Calculated energy variation δE for the interlayer excitons in the ON (**b** free diffusion, *V*_g1_ = *V*_g2_ = *V*_g3_ = 0 V) and OFF (**c** potential barrier, *V*_g1_ = 16 V, *V*_g2_ = *V*_g3_ = 0 V) states^29^. Red arrows represent laser excitation, and black dashed arrows denote interlayer exciton diffusion. **d**, **e** Corresponding images of the interlayer exciton emission in the ON and OFF states^29^. Dashed lines denote the positions of the MoS_2_ and WSe_2_ monolayers and the top graphene gate (gate 1). The red circle represents the laser spot. Scale bars, 5 μm. **f** Calculated energy profile δE of the interlayer exciton as a function of the lateral coordinate *X* under the forward bias case (*V*_g1_ = 0 V, *V*_g2_ = 5 V, *V*_g3_ = 10 V)^29^. The black solid lines show the direction of interlayer exciton drift. **g** Image of the interlayer exciton emission under the forward bias in **f**, demonstrating a drift distance of ~5 μm^29^. Scale bars, 5 μm. **h**, **i** Calculated energy profile δE of the interlayer exciton for the cases of a potential well (**h** confinement) and a potential barrier (**i**, expulsion)^29^. **j**, **k** Images of the interlayer exciton emission for the potential landscapes shown in **h** and **i**^29^. Scale bars, 5 μm. **a**–**k** Reprinted with permission from ref. ^29^ [Springer Nature Limited]

The above investigations focused on interlayer exciton transport at low temperature, while practical applications prefer devices operating at room temperature. The upper bound of the temperature (*T*) where excitons can exist can be roughly determined by *E*_b_/*k*_B_, where *E*_b_ is the exciton binding energy and *k*_B_ is the Boltzmann constant^172,186,187^. In traditional semiconductor CQWs, the resulting indirect excitons usually have a small *E*_b_ of several to tens of meV (<*k*_B_*T* in most cases), resulting in excitonic devices generally operating at cryogenic temperatures^172,187,188^. However, the interlayer excitons formed in TMD vdW heterostructures have a much larger *E*_b_, with reported values exceeding 100 meV^17,21,42,55,87,81–86,88,89^, leading to a much higher predicted temperature (>1000 K), which facilitates the realization of excitonic devices that operate at room temperature. In fact, excitonic devices based on an hBN-encapsulated MoS_2_/WSe_2_ heterobilayer with electrically controlled transistor actions at room temperature were realized recently^29^. In the vertical direction of this heterostructure, multiple transparent graphene electrodes (gates 1–3, gate voltages *V*_g1_–*V*_g3_) were fabricated to provide a laterally modulated potential landscape to control the exciton flux (Fig. 7a)^29^. In the absence of electric fields (*V*_g1–g3_ = 0), the interlayer excitons could freely diffuse away from the laser spot by approximately 3 μm and reach the recombination site with a bright emission output (marked as the “ON” state for the excitonic switch, Fig. 7b, d). Note that during this diffusion process, the interlayer excitons were expected to dissociate into single carriers that could diffuse inside monolayer WSe_2_ and MoS_2_ to recombine with the native charges^29^. However, when introducing a potential barrier higher than *k*_B_*T* on the diffusion path (i.e., *V*_g1_ = +16 V, *V*_g2,__g3_ = 0), the interlayer exciton transport was impeded by the barrier, causing a dark output (marked as the “OFF” state for the excitonic switch, Fig. 7c, e). Therefore, a change in the ON and OFF states in the excitonic switch could be realized by selectively introducing a barrier to either allow or block exciton transport. The intensity ratio of the ON and OFF states was reported to be larger than 100 at room temperature. In addition, an upwards or downwards potential gradient could be laterally created by using all the electrodes to further manipulate the exciton transport. For instance, the interlayer exciton transport could be enhanced (over 5 μm) under a progressively lower energy profile due to the addition of drift motion along the path (Fig. 7f, g). Moreover, various types of potential landscapes, such as a potential well (Fig. 7h, j) or a repulsive barrier (Fig. 7i, k), could be created by applying appropriate voltages to either trap the interlayer excitons in the well or allow them to drift away from the barrier, opening a route to versatilely manipulate the exciton transport in future excitonic devices. Room-temperature exciton transport was also found in another MoS_2_/WSe_2_ heterobilayer stacked on a silicon-on-insulator substrate with a silicon suspended slab in the middle^136^. Intriguingly, this unique suspended slab geometry introduced a strong potential gradient for the interlayer excitons, combined with their long recombination lifetimes, both of which enabled the observation of the valley Hall effect even at room temperature^136^.

### Valley-polarized interlayer exciton transport

**Fig. 8:**
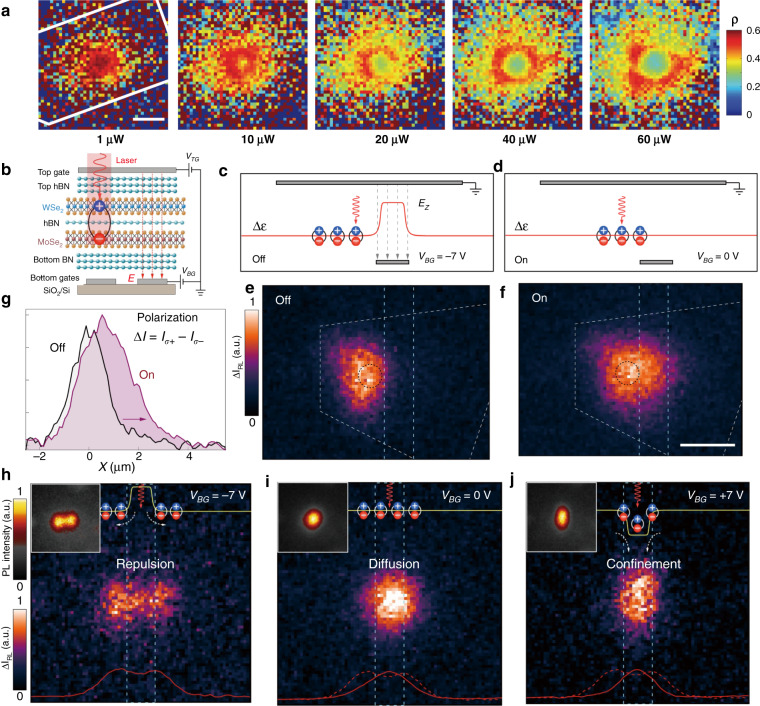
Valley-polarized interlayer exciton transport. **a** Spatial map of valley polarization (*ρ*) of the interlayer exciton emission under 1 to 60 μW excitation^33^. The white outline represents the sample region. Scale bar, 2 μm. **b–j** Valley polarized interlayer exciton transport controlled by external potential landscapes^34^. **b** Schematic illustration of the interlayer exciton transistor with switch functions achieved by controlling the external gate voltages. **c**, **d** Numerically simulated energy profile (red line) of interlayer excitons in the OFF (**c**, a potential barrier, *V*_TG_ = 0 V, *V*_BG_ = − 7 V) and ON (**d**, free diffusion, *V*_TG_ = 0 V, *V*_BG_ = 0 V) states of the excitonic transistor. **e**, **f** Real-space CCD images of the interlayer exciton polarization (Δ*I*_RL_) in the OFF and ON states shown in **c** and **d**. Δ*I*_RL_ = *I*_σ+_ – *I*_σ−_ is the difference between the σ^+^ and σ^−^ circularly polarized emission intensities of the interlayer excitons. **g** Intensity profiles of the emitted polarization along a cutline in the middle of **e** and **f** [ref]. Scale bars, 2 μm. **h**–**j** Real-space CCD images of the interlayer exciton polarization corresponding to the potential landscape configurations of repulsion (**h**), diffusion (**i**), and confinement (**j**). The simulated energy profile for the interlayer excitons is shown as a yellow line in the three cases. The red line shows the intensity profile along the lateral direction in the middle of the image. The red intensity profile in **h** is replicated as a dashed line in **i** and **j**. Insets show the PL intensity images. **a** Reprinted with permission from ref. ^33^ [American Association for the Advancement of Science]. **b**–**j** Reprinted with permission from ref. ^34^ [Springer Nature Limited]

Compared to the indirect excitons formed in traditional semiconductor CQWs, another advantage of the interlayer excitons in TMD vdW heterostructures is their inherited valley-contrasting physics. As discussed before, interlayer excitons have long valley-polarized lifetimes as well, ranging from nanoseconds to microseconds^27,33^, which has motivated scientists to explore the transport properties of valley-polarized interlayer excitons. An early work demonstrated that the lateral drift and diffusion of valley-polarized interlayer excitons could reach several micrometers owing to their long valley-polarized lifetimes (~40 ns)^33^. The spatial pattern of the valley polarization for the interlayer excitons evolved into a ring with a diameter that increased with the excitation intensity (Fig. 8a)^33^, which was strikingly different from the spatial pattern of unpolarized interlayer excitons stated above. Since exciton diffusion due to a density gradient is valley-independent and unlikely to generate a ring pattern, the observed ring pattern in the valley polarization was ascribed to valley-dependent exciton exchange interactions that caused the majority valley excitons to experience a stronger repulsive force, and thus more rapid expansion than the minority excitons leading to valley-asymmetric transport of the interlayer excitons with the resulting ring pattern in the spatial distribution^33,35^.

Similar to the aforementioned excitonic switch based on the manipulation of interlayer exciton transport, an excitonic switch based on valley-polarized interlayer excitons was also recently realized at low temperatures (4 and 100 K)^34^. The ON and OFF states of this switch were achieved by removing and introducing a potential barrier on the transport path, and an approximately 1.4 µm difference in the diffusion distance of the valley-polarized interlayer excitons was observed between the ON and OFF states (Fig. 8b–g)^34^. Analogously, the transport behavior (repulsive drift or confinement trap) of the valley-polarized interlayer excitons could be manipulated by designing various potential profiles, such as a potential barrier or well along the travel path (Fig. 8h–j). Very interestingly, when a potential well was applied to trap valley-polarized interlayer excitons, the exciton concentration could be increased by an order of magnitude (a lower bound of ~1.8 × 10^12^ cm^−2^), which was a promising step towards the realization of a macroscopic quantum state of valley-polarized excitons via Bose–Einstein condensation^34^. Further work is expected to achieve the accumulation of valley-polarized excitons at higher temperatures by engineering or optimizing the potential profiles in these systems.

Notably, single particles such as resident holes in hole-doped heterostructures can serve as efficient valley information carriers as well, with a reported spin-valley diffusion current persisting for more than 20 μs accompanied by a travel distance of over 20 μm^170^. A detailed description of this topic is beyond the scope of this review.

### Interlayer exciton transport under moiré potentials

In TMD vdW heterostructures, the potential landscape for manipulating the interlayer exciton transport can be modified not only by external electric fields but also by an internal moiré pattern, which introduces a twist-angle-dependent moiré potential on the interlayer excitons. Thus far, the above discussed interlayer exciton transport has focused on the exciton motion under externally applied electric fields. Experimental investigations on how excitons move in a potential landscape modulated by moiré patterns are rare.

**Fig. 9:**
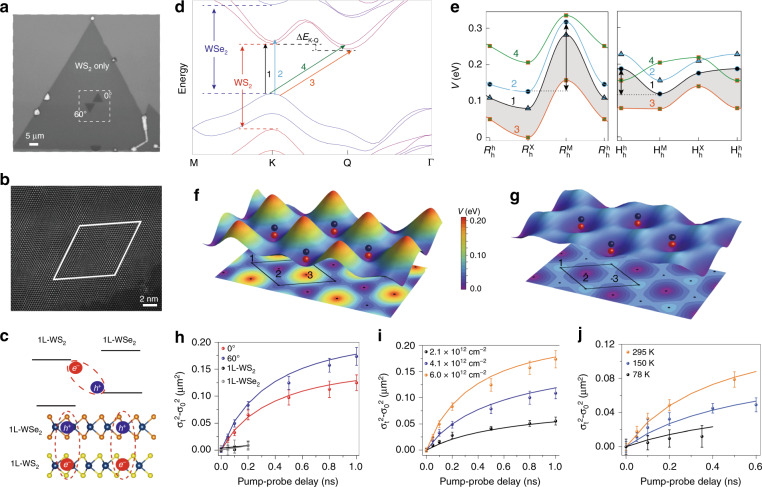
Interlayer exciton transport under a moiré potential. **a** Optical image of two CVD-grown WS_2_/WSe_2_ heterobilayers with twist angles of 0 and 60° on the same WS_2_ underlayer^91^. **b** High-resolution annular dark-field scanning transmission electron microscopy image of a 60° heterobilayer^91^. The white diamond outline shows a moiré superlattice with a periodicity of ~7.6 nm. **c** Schematic illustration of the WS_2_/WSe_2_ heterobilayer with a type-II band alignment for facilitating interlayer exciton formation^91^. **d** Schematic representation of the typical electronic band structure of a WS_2_/WSe_2_ heterobilayer in a (strained) primitive unit cell^91^. The four lowest-energy transitions are indicated by arrows (K–K valley transitions are denoted by vertical arrows 1 and 2, and K–Q valley transitions are denoted by vertical arrows 3 and 4). The K–K transitions in individual WS_2_ and WSe_2_ monolayers are marked by vertical arrows WS_2_ and WSe_2_, respectively. **e** Approximate moiré potentials for twist angles of 0° (left) and 60° (right) plotted along the main diagonal of the moiré supercells (black lines in **f**)^91^. The spatial potential variations for 0° are much stronger (deep potential) than those for 60° (shallow potential) heterobilayers. The different lines correspond to the four lowest-energy optical transitions marked in **d**. **f**, **g** Illustrations of both 3D graphs and 2D projections of the 2D K-K moiré potentials for trapping interlayer excitons (red and black spheres) in local minima for 0° (**f**) and 60° (**g**) heterobilayers^91^. A twist-angle-dependent moiré potential is indicated by the theoretical results. The numbers 1, 2, and 3 indicate the three high-symmetry local atomic registries in a moiré superlattice. **h** Time-dependent mean squared distances (*σ*_t_^2^ − *σ*_0_^2^) traveled by interlayer excitons in 0° and 60° heterobilayers as well as by intralayer excitons in WS_2_ and WSe_2_ monolayers (1L-WS_2_, 1L-WSe_2_)^91^. **i** Density-dependent interlayer exciton transport at room temperature for the 60° heterobilayer^91^. **j** Temperature-dependent interlayer exciton transport for the 60° heterobilayer^91^. **a**–**j** Reprinted with permission from ref. ^91^ [Springer Nature Limited]

Recently, the role of moiré potentials in exciton transport was experimentally addressed using transient absorption microscopy (TAM) combined with first-principles calculations^91^. In this study^91^, WS_2_/WSe_2_ heterobilayers with two different twist angles (0 and 60°) were fabricated to study the effect of the twist-angle-dependent moiré potential on exciton transport (Fig. 9a–c). The moiré potentials of 0° and 60° heterobilayers were first theoretically calculated (Fig. 9d–g), with the spatial variations for 0° being much stronger (deep potential) than those for 60° (shallow potential), implying that the interlayer excitons encountered a higher energy barrier as they moved from one location to another in the 0° heterobilayers. In other words, the interlayer excitons in 60° heterobilayers should be more mobile due to less confinement from the shallow potential. This was further proven by imaging the spatiotemporal-dependent exciton population via the TAM technique, from which the time-dependent mean squared distances ($\sigma _{\rm{t}}^2 - \sigma _0^2$) traveled by both inter- and intralayer excitons were obtained (Fig. 9h). Deviating from the normal diffusion behavior of intralayer excitons with a linear temporal dependence on the mean squared distance, anomalous diffusion of interlayer excitons, with a nonlinear temporal dependence on the mean squared distance and a faster diffusion speed, was observed for both 0° and 60° heterobilayers, suggesting that the repulsive interlayer exciton interactions played a central role in their diffusion process. As expected, the interlayer excitons in 60° heterobilayers traveled faster than those in 0° heterobilayers, consistent with the prediction from calculations and strongly indicating the role of moiré potentials in mediating exciton transport. The interlayer excitons were also transported faster at higher excitation densities owing to the repulsive interactions between the interlayer excitons (Fig. 9i).

The effect of moiré potentials on exciton transport was further supported by temperature-dependent measurements. As observed, the exciton transport speed increased as the temperature increased (Fig. 9j)^91^ owing to the more effective screening of the moiré potentials at higher temperatures. This observation is seemingly inconsistent with the temperature-dependent steady-state PL measurements, with the interlayer excitons transported over longer distances at lower temperatures^29,30,34^. However, steady-state measurements are integrated over the entire exciton lifetime, and the slower exciton transport at lower temperatures could be compensated by the corresponding longer lifetimes, which could lead to an overall longer transport distance at lower temperatures^91^.

**Fig. 10:**
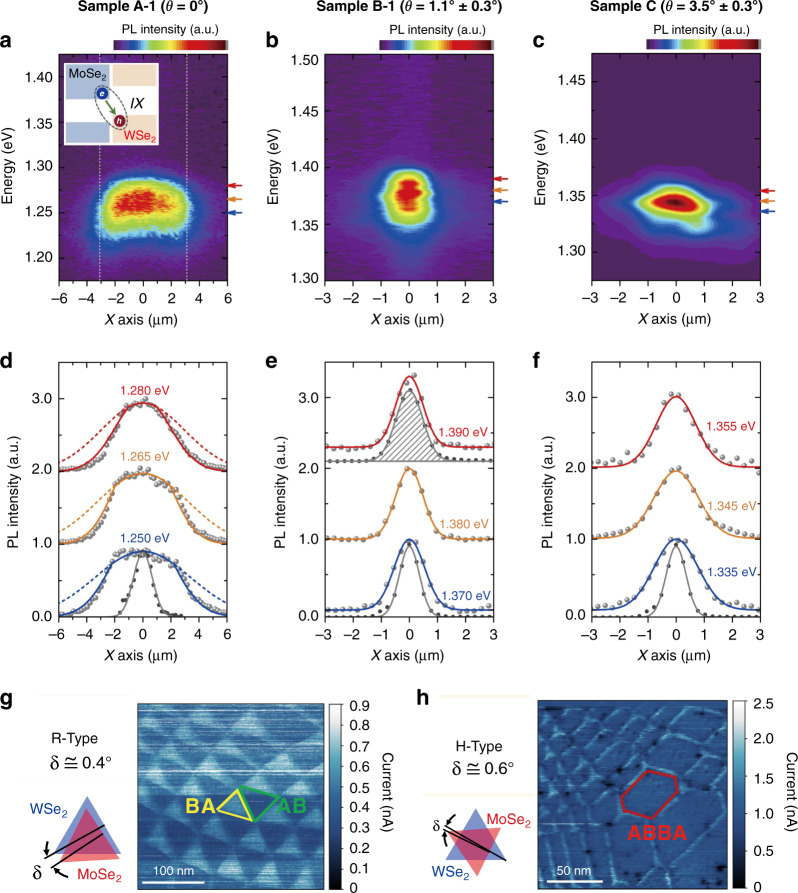
Interlayer exciton diffusion at different twist angles. **a**–**c** Spatially resolved PL images of interlayer excitons in MoSe_2_/WSe_2_ heterostructures prepared by the CVD growth method (**a** sample A-1) and mechanical exfoliation and transfer method with a twist angle of 1.1° ± 0.3° (**b** sample B-1) and a twist angle of 3.5° ± 0.3° (**c** sample C)^162^. **d**–**f** PL line profiles extracted from **a**–**c** for the three samples at a few selected energies. The PL line profiles for sample A-1 are truncated by the boundaries of the heterostructure region (the white dashed lines in **a**)^162^. The 660 nm excitation laser profile and the 900 nm laser profile are represented by the bottom gray lines and the top gray shaded area, respectively. The PL spot size of the interlayer exciton (PL wavelength near 900 nm) shown in Fig. 10e is slightly larger than the excitation laser (660 nm) spot size because of the imaging optics at different wavelengths. A Gaussian function was used to fit the measured data points. **g** Atomic reconstruction in the R-type MoSe_2_/WSe_2_ heterostructure with a small twist angle (*δ*) of ~0.4°^194^. The yellow and green triangles represent the triangular domains with BA and AB stacking, respectively. **h** Atomic reconstruction in the H-type MoSe_2_/WSe_2_ heterostructure with a small twist angle (*δ*) of ~0.6°^194^. The red hexagon represents the hexagonal domain with ABBA stacking. **a**–**f** Reprinted with permission from ref. ^162^ [American Association for the Advancement of Science]. **g**, **h** Reprinted with permission from ref. ^194^ [American Chemical Society]

Similar to the former discussed work, interlayer exciton transport under periodic moiré potential in both R-type and H-type MoSe_2_/WSe_2_ heterostructures was studied later, from which the diffusion barrier experienced by the interlayer excitons was experimentally quantified by studying the exciton transport at various temperatures^189^. The influence of the moiré potential on interlayer exciton diffusion was also studied recently via spatially and spectrally resolved PL imaging in MoSe_2_/WSe_2_ heterostructures with different twist angles (Fig. 10a–f)^162^. In this study, three types of heterostructures were used, with the first prepared by the CVD method with a twist angle of 0° (no moiré pattern) and the second and third prepared by the mechanical exfoliation and transfer (MET) method with twist angles of ~1.1° (moiré period of ~17 nm) and ~3.5° (moiré period of ~5 nm), respectively. The interlayer exciton diffusion of these samples was remarkably different (Fig. 10a–f). The diffusion length in the CVD-grown sample with no moiré potential was the longest (a few microns), exceeding the boundaries of the heterostructure region. The diffusion in the heterostructure with a twist angle of ~3.5° (a moiré period of ~5 nm) was finite, with a diffusion length of ~1 µm, while no diffusion beyond the laser spot was observed in the heterostructure with a relatively smaller twist angle (~1.1°) but a larger moiré period (~17 nm). These results suggested that a moiré superlattice has a profound effect on interlayer exciton diffusion. As stated^162^, diffusion in CVD-grown commensurate heterostructures with no moiré pattern should be the most free and reach the longest distance. In contrast, in heterostructures with a larger moiré period (a smaller twist angle), the interlayer excitons could be substantially localized by the moiré potential without obvious diffusion. For heterostructures with a smaller moiré period (a larger twist angle and a longer interlayer exciton lifetime due to the larger momentum mismatch), interlayer excitons may tunnel between the supercells and diffuse a finite distance over a longer interlayer exciton lifetime. All these works indicate that the diffusion barrier introduced by the moiré potential could be modified to affect exciton migration by tuning the twist angle or stacking mode between the constituent monolayers, which offers a novel way to control the exciton transport behavior in potential excitonic devices.

However, note that when the twist angle between layers was rather small (generally less than 1°), atomic reconstruction could take place and was expected to have a profound effect on the band structures of the twisted bilayers^190–193^. In this case, the continuously varying rigid-lattice moiré pattern transformed to discrete commensurate domains divided by narrow domain walls, which had also been recently observed in twisted TMD vdW bilayers^194,195^. As reported^194^, in R-type heterostructures with a small twist angle of <1°, atomic reconstruction could induce commensurate triangular domains with alternating AB and BA stacking configurations dominating in the whole heterostructure region (AB: transition metal A in the upper layer overlaps chalcogen B in the bottom layer; BA: chalcogen B in the upper layer overlaps transition metal A in the bottom layer), while in H-type heterostructures with a twist angle of <1°, atomic reconstruction resulted in commensurate hexagonal domains dominated by the ABBA stacking configuration (ABBA: transition metal A in the upper layer overlaps chalcogen B in the bottom layer and chalcogen B in the upper layer overlaps transition A in the bottom layer). The average area of the hexagonal domains in the H-type system was reported to approach twice that of the triangular domains in the R-type system^194^. In other words, for atomically reconstructed bilayers with small twist angles, the high-symmetry stacking configurations that only occurred at certain sites in the rigid-lattice moiré pattern now covered almost the whole bilayer region, either in alternating AB and BA stacking (R-type) or in ABBA stacking (H-type), which was reported to have lower stacking energies^194^, with their boundaries separated by very narrow domain walls (several nm^194,195^). Within these enlarged commensurate domains (several tens of nm or even larger than 100 nm^194,195^), the moiré potential was absent, although a diffusion barrier may have existed between the boundaries. Therefore, the potential landscape imposed on the interlayer excitons of a moiré heterostructure with a relatively large twist angle may vary notably from those of an atomic reconstructed heterostructure with a very small twist angle. Correspondingly, the scenarios of the interlayer exciton transport may be different for these two classes of heterostructures. These differences may even exist between the R-type and H-type heterostructures after atomic reconstruction due to their remarkably different rearranged configurations. Further theoretical and experimental investigations are expected to reveal these discrepancies and to provide deep insight into this new type of heterostructure.

## Applications

After a detailed description of the interlayer exciton formation, relaxation, and transport properties in TMD vdW heterostructures, a brief introduction of its potential applications is presented in this section. According to the above discussion, interlayer excitons have a number of excellent characteristics such as (1) high exciton binding energy (>100 meV), (2) energy tuneable by an external electric field, (3) long population and valley-polarized lifetimes, (4) long-range transport, and (5) the potential to realize high-temperature Bose–Einstein condensates. All these factors make interlayer excitons very suitable for developing excitonic devices as the counterpart of electronic devices for signal processing^173^, and such excitonic signal processing devices have the following advantages: first, interlayer excitons and photons can directly transform into each other, allowing efficient coupling of the signal processing to optical communication, especially in potential silicon-based optoelectronics; second, interlayer excitons can act as spin–valley information carriers due to the inherited spin–valley properties, enabling efficient signal processing with reduced power consumption; and third, interlayer excitons hold great promise for realizing a high-temperature coherent condensate^32^, making the creation of an exciton current with no resistance possible in future computation.

**Fig. 11:**
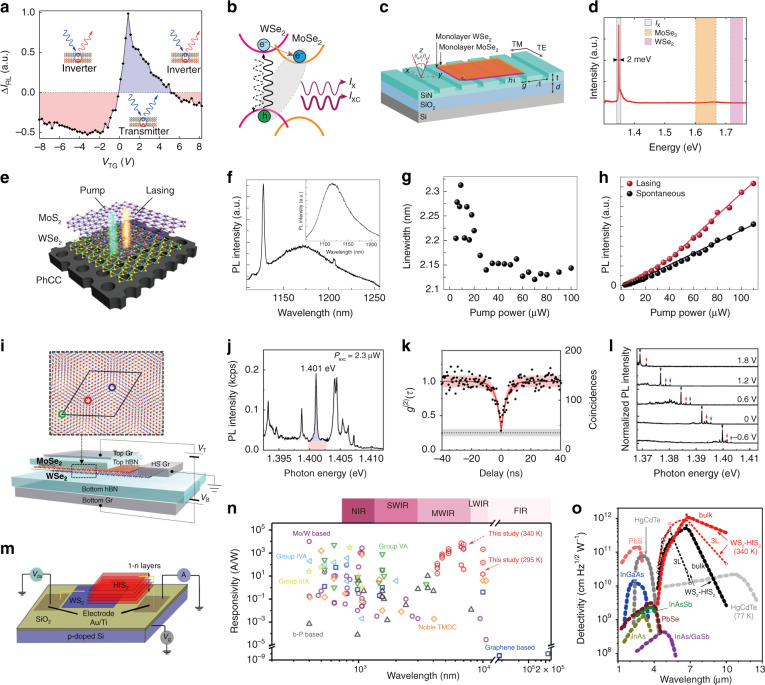
Excitonic devices based on interlayer excitons. **a** Polarization switching actions based on the interlayer excitons in a MoSe_2_/WSe_2_ heterostructure^117^. Δ*I*_RL_ = *I*_R_ − *I*_L_ is the difference between the right (*I*_R_) and left (*I*_L_) circularly polarized emission intensities of the interlayer excitons. *V*_TG_ is the gate voltage. **b** Type-II band alignment of the TMD vdW heterobilayer, forming a three-level system for lasing^196^. **c** Schematic illustration of a laser device with a MoSe_2_/WSe_2_ heterobilayer integrated in a silicon nitride grating resonator^196^. **d** Interlayer exciton lasing from the device in **c** at 5 K^196^. The shaded boxes represent the spectral range of interlayer (*I*_X_), MoSe_2_, and WSe_2_ exciton emission. **e** Schematic illustration of the fabricated MoS_2_/WSe_2_ heterobilayer-PhCC nanolaser^96^. PhCC photonic crystal cavity. **f** Interlayer exciton lasing from the device in **e** at room temperature^96^. The linewidth is ~2.26 nm. The inset shows the spontaneous emission of the interlayer exciton for comparison. **g** Linewidth of the interlayer exciton emission as a function of the pump power for the laser device in **e**^96^. **h** Light input–light output (L–L) curve showing the cavity interlayer exciton emission from the device in **e** with a kink, suggesting the onset of superlinear emission and lasing operation^96^. The spontaneous emission displays a linear dependence on the pump power. **i** Schematic illustration of the MoSe_2_/WSe_2_ moiré heterostructure with a moiré pattern for the realization of quantum emitters^142^. **j** PL spectrum of the moiré-trapped interlayer excitons formed in the MoSe_2_/WSe_2_ moiré heterostructure at 4 K^142^. **k** The second-order correlation function *g*^(2)^(*τ*) of a single emitter at 1.401 eV shown in **j**^142^. **l** Stark tuning of moiré-trapped interlayer excitons at different gate voltages^142^. **m** Schematic illustration of the interlayer exciton photodetector based on the WS_2_/HfS_2_ heterostructure on a doped silicon substrate^197^. **n** The peak responsivity of the interlayer exciton photodetector based on the WS_2_/HfS_2_ heterostructure (*V*_g_ = 0 V, *V*_ds_ = −1.5 V and *I*_device_ = 0.5 nW) and that of other reported 2D-based photodetectors in the visible and infrared range^197^. NIR near infrared, SWIR short-wavelength infrared, MWIR mid-wavelength infrared, LWIR long-wavelength infrared, FIR far infrared. **o** Specific detectivity as a function of wavelength for WS_2_/HfS_2_ photodetectors shown in **i** and the other commercially available photodetectors at room temperature, except the one at 340 K^197^. **a** Reprinted with permission from ref. ^117^ [Springer Nature Limited]. **b**–**d** Reprinted with permission from ref. ^196^ [Springer Nature Limited]. **e**–**h** Reprinted with permission from ref. ^96^ [American Association for the Advancement of Science]. **i**–**l** Reprinted with permission from ref. ^142^ [American Association for the Advancement of Science]. **m**–**o** Reprinted with permission from ref. ^197^ [Springer Nature Limited]

Various excitonic signal processing devices such as excitonic transistors, routers, and photon storage devices have been explored based on indirect excitons in traditional semiconductor CQWs^173^, which motivates investigations of excitonic devices based on interlayer excitons in TMD vdW heterostructures. As stated above, excitonic transistors with switch functions from the manipulation of the interlayer exciton transport have been achieved by proof-of-principle in TMD vdW heterostructures^29,34^. An excitonic switch through exchange of the emission polarization of interlayer excitons has also been recently demonstrated in a MoSe_2_/WSe_2_ heterostructure^117^. The difference between right and left circularly polarized emission intensities (Δ*I*_RL_) as a function of the gate voltage (*V*_TG_) was studied in this work to demonstrate the polarization switching actions (Fig. 11a)^117^. For *V*_TG_ below 0 V or higher than 5 V, an “inverter” reversing the input polarization (negative Δ*I*_RL_) was obtained. However, for *V*_TG_ between 0 and 5 V, a “transmitter” preserving the input polarization (positive Δ*I*_RL_) could be achieved. Such electrical manipulation of the emission polarization of interlayer excitons paves the way for their practical applications in signal processing and integrated optoelectronics.

In addition to signal processing devices, other devices, especially light sources and photodetectors, are keystone components in integrated optoelectronics or silicon-based optoelectronics. Very encouragingly, excitonic lasers and photodetectors based on interlayer excitons were recently realized in TMD vdW heterostructures^96,196,197^. Owing to the ultrafast charge transfer and the long lifetimes of the formed interlayer excitons, the type-II band aligned heterostructure actually provides a three-level system to facilitate the establishment of a population inversion for lasing (Fig. 11b)^196^. Through this, spatially coherent lasing emission from direct-bandgap interlayer excitons was realized in a rotationally aligned MoSe_2_/WSe_2_ heterobilayer integrated in a silicon nitride grating resonator at low temperature (Fig. 11c, d)^196^. Later, a room-temperature near-infrared laser based on interlayer excitons in a MoS_2_/WSe_2_ heterobilayer on a silicon photonic crystal cavity was achieved (Fig. 11e–h)^96^. These works indicate that interlayer excitons in TMD vdW heterostructures can act as an efficient gain medium to support lasing emission, which may offer new opportunities for developing coherent light sources with desired optical properties for integrated optoelectronics. Moreover, quantum light based on moiré-trapped interlayer excitons in a MoSe_2_/WSe_2_ heterostructure has been reported recently (Fig. 11i–l)^142^, although the emission from a single moiré unit cell was not specified. In this study, the moiré-trapped interlayer excitons manifested as several discrete sharp lines in the PL spectrum, with extraordinarily narrow linewidths of several tens to hundreds of µeV (Fig. 11j). The quantum nature of the moiré-trapped interlayer excitons was established via the observation of photon antibunching (Fig. 11k). In addition, the emission energy of these quantum emitters could be tuned up to 40 meV through the direct current Stark effect (Fig. 11l). This study provides a route for investigating and understanding the nature of moiré quantum emitter arrays. On the other hand, photodetectors, serving as optical to electrical converters, are also an essential component of integrated optoelectronics. A highly responsive room-temperature-operated infrared photodetector, with detection range tuning from mid-wavelength to long-wavelength infrared (MW-LWIR) under a modest electric field, was realized recently based on interlayer excitons in a WS_2_/HfS_2_ heterostructure (Fig. 11m)^197^. The responsivity of this MW-LWIR photodetector was reported to be comparable to those of Mo/W-based photodiodes in the visible and near infrared range (Fig. 11n), and the calculated detectivities (D*) of this photodetector were shown to be higher than those of other commercially available infrared photodetectors, especially for room and elevated temperature operations (Fig. 11o). These findings demonstrate the great promise of interlayer excitons for versatile optoelectronic applications.

Nevertheless, research on excitonic devices based on interlayer excitons in TMD vdW heterostructures is still in the early stages. Improving the performance of the already developed excitonic devices for practical applications and exploring more functional excitonic devices, such as waveguides and modulators are expected in further works. Moreover, the integration of individual excitonic devices such as light sources, switches, modulators, and detectors on a single chip is very likely and highly desirable in the future for realizing on-chip integrated optoelectronics based on two-dimensional vdW heterostructures^96,198^.

## Conclusions and outlook

The rise of TMD vdW heterostructures has stimulated enormous interest in interlayer excitons, including their formation, relaxation, transport, and related applications. All these research topics are closely linked to each other, as discussed above. Specifically, the ultrafast charge transfer in type-II-band-aligned TMD vdW heterostructures facilitates the formation of interlayer excitons, which have a variety of fundamental characteristics such as the existence of two different kinds of electric dipoles (a reduced transition dipole and a permanent electric dipole), inherited valley-contrasting physics, and moiré configuration dependence. Benefiting from these fundamental properties, the interlayer excitons have a long population lifetime and possess significantly suppressed intervalley scattering, both of which give rise to a long valley-polarized lifetime for the interlayer excitons. As a consequence, the interlayer excitons and valley-polarized interlayer excitons have good transport characteristics, basically owing to their long lifetimes and sensitivity to external electric fields. All these factors form the basis for developing excitonic optoelectronic devices with interlayer excitons, such as excitonic transistors^29,34^, switches^117^, lasers^96,196^, and photodetectors^197^.

Undoubtedly, as a newly controllable excitonic system, the interlayer excitons in TMD vdW heterostructures offer a very promising platform for both fundamental exploration and potential optoelectronic applications. For fundamental studies, beyond the rapid progress in revealing the intriguing features of interlayer excitons, overcoming the existing obstacles and discovering new sciences are expected to further push this area towards the central stage. For instance, a comprehensive understanding of the underlying mechanism responsible for ultrafast charge transfer with conserved spin–valley properties will lay the basis for fully clarifying the subsequent interlayer exciton formation process as well as its inherited valley-contrasting physics, which plays an important role in determining the degree of its valley polarization. Additionally, significant efforts are needed to thoroughly comprehend the valley depolarization mechanism and its relationship with the constituted materials, crystallographic alignment, stacking modes, doping, and external fields or potentials, aiming to unravel the interplay of these factors and achieve a sufficiently high degree of valley polarization at room temperature for practical valleytronic applications. The origins of the observed negative circular polarization and doublet interlayer excitons are also an open questions, that remain to be addressed for an in-depth understanding of the nature of interlayer excitons. Moreover, interlayer excitons offer a novel and very exciting platform for exploring excitonic Bose condensation owing to their substantial binding energies, which stabilize them at relatively high temperature; their sufficiently long lifetimes for cooling them to approach the lattice temperature; and the existing oriented or permanent electric dipoles with repulsive interactions, which prevent the formation of competing exciton complexes at high densities^35,199,200^. All these merits are expected to facilitate the realization of an excitonic condensate and/or superfluidity at high temperature based on interlayer excitons or even valley-polarized interlayer excitons^31,32^. Another intriguing topic for future exploration is moiré interlayer excitons, which have recently attracted enormous interest. Owing to their interlayer configurations, the spatially indirect interlayer excitons are expected to be significantly influenced by moiré patterns that carry periodically varied interlayer atomic registries. However, how the interlayer excitons and their properties are spatially modulated by the moiré pattern or potential remains to be intensively explored. Far-field optical spectroscopy has been applied to reflect the moiré effect on interlayer excitons^91,141,150,152,153,155^, while near-field optics, with a spatial resolution sufficient to resolve the intersite in a moiré superlattice, will be the more intuitive tool for selectively addressing the interlayer excitons at individual sites. In addition, it is important to know in which situations the moiré effect is favorable and in which cases it should be avoided. For instance, the moiré potential plays an important role in trapping the interlayer excitons for quantum optics^36^, whereas it turns to be an obstacle for driving interlayer exciton transport^91^. When the moiré lattice needs to be avoided in some applications, how to avoid it while preserving the other advantages of interlayer excitons is quite important. One possible solution is to use commensurate heterobilayers prepared by continuous growth via the CVD technique^114,137,162,201^.

For potential optoelectronic applications, as stated above, various excitonic devices such as lasers^96,196^, transistors^29,34^ and photodetectors^197^ have been realized by proof-of-principle based on interlayer excitons in TMD vdW heterostructures. Specifically, light sources such as lasers based on interlayer excitons have great potential in silicon photonics and optical fiber communication systems due to the following aspects: (1) the heterostructures formed with van der Waals forces could remarkably ease the problem of lattice mismatch that restricts the material growth and integration; (2) the interlayer exciton emission wavelength is tuneable by either the constituted materials or external electric field to be compatible with the silicon photonics or optical fiber communication band; and (3) the use of the type-II band alignment to form the interlayer excitons provides a natural three-level system, providing the possibility of population inversion for achieving coherent light sources. Such advantages promise a bright future for interlayer excitons in photonic applications. More efforts will be dedicated to improving the performance of the developed light sources such as increasing the absorption/emission of the interlayer excitons by coupling with plasmonics, creating hybrid interlayer excitons with inherited brightness from the intralayer counterparts, and designing multilayer stacking modes to increase both the interlayer coupling and the absorbance. Additionally, electrically pumped light sources will be highly desirable and will very likely be realized with interlayer excitons for integrated optoelectronic applications. Similar strategies could be applied for valley-polarized interlayer excitons to realize polarized light sources carrying valley information. Further, quantum emitters based on interlayer excitons will be a promising direction for future exploration. Various potential minima may be constructed by electric fields, strains, or especially moiré patterns to trap either one or two interlayer excitons^142^, enabling the realization of single photon sources or entangled-photon sources for potential quantum computation and information processing. Other excitonic devices such as transistors operating in switching mode have been reported with interlayer excitons^29,34^, while such devices operating in amplification mode are still lacking and highly desirable for practical signal processing. Moreover, more functional excitonic devices such as waveguides and modulators await exploitation to further extend and promote practical applications based on interlayer excitons. More intriguingly, it is expected that the full integration of these individual excitonic devices, such as light sources, waveguides, modulators, and detectors, on a single chip to realize on-chip integrated optoelectronics based on interlayer excitons will be highly possible in the future.
